# Population genomics and history of speciation reveal fishery management gaps in two related redfish species (*Sebastes mentella *and *Sebastes fasciatus*)

**DOI:** 10.1111/eva.13143

**Published:** 2020-12-14

**Authors:** Laura M. Benestan, Quentin Rougemont, Caroline Senay, Eric Normandeau, Eric Parent, Rick Rideout, Louis Bernatchez, Yvan Lambert, Céline Audet, Geneviève J. Parent

**Affiliations:** ^1^ CEFE Univ Montpellier, CNRS, EPHE‐PSL University IRD, Univ Paul Valéry Montpellier 3 Montpellier France; ^2^ Département de biologie, Institut de Biologie Intégrative et des Systèmes (IBIS) Université Laval Québec QC Canada; ^3^ Fisheries and Oceans Canada Maurice‐Lamontagne Institute Mont‐Joli QC Canada; ^4^ Fisheries and Oceans Canada Northwest Atlantic Fisheries Centre N.L. St. John’s Canada; ^5^ Institut des sciences de la mer de Rimouski Université du Québec à Rimouski Rimouski QC Canada

**Keywords:** demographic models, fishery management, population genomics, related species, *Sebastes*, spatial ecology

## Abstract

Understanding the processes shaping population structure and reproductive isolation of marine organisms can improve their management and conservation. Using genomic markers combined with estimation of individual ancestries, assignment tests, spatial ecology, and demographic modeling, we (i) characterized the contemporary population structure, (ii) assessed the influence of space, fishing depth, and sampling years on contemporary distribution, and (iii) reconstructed the speciation history of two cryptic redfish species, *Sebastes mentella* and *S. fasciatus*. We genotyped 860 individuals in the Northwest Atlantic Ocean using 24,603 filtered single nucleotide polymorphisms (SNPs). Our results confirmed the clear genetic distinctiveness of the two species and identified three ecotypes within *S. mentella* and five populations in *S. fasciatus*. Multivariate analyses highlighted the influence of spatial distribution and depth on the overall genomic variation, while demographic modeling revealed that secondary contact models best explained inter‐ and intragenomic divergence. These species, ecotypes, and populations can be considered as a rare and wide continuum of genomic divergence in the marine environment. This acquired knowledge pertaining to the evolutionary processes driving population divergence and reproductive isolation will help optimizing the assessment of demographic units and possibly to refine fishery management units.

## 1 INTRODUCTION

Delineating sustainable management boundaries through the characterization of genomic variation among and within species can provide an indication of reproductive isolation and ultimately help to reduce the risk of depletion of marine resources (Laikre et al., 2009; Ovenden et al., 2015; Reiss et al., 2009). Yet, this represents a challenging endeavor since it reflects both contemporary and historical processes (Waples and Gaggiotti, 2006). It has now become feasible to document these two processes occurring at different timescales by combining population genomics, spatial ecology, and demographic model approaches. Together, these three approaches can identify how species, ecotypes, and populations diverged through space and time, which ultimately lead to more suitable recommendations for the management and conservation of commercially valuable stocks. Population genomics allows detecting and separating locus specific from genome‐wide effects to improve our understanding of evolution (Black et al., 2001). Population genomics information can then be used in spatial ecology to quantify the drivers that may explain genomic distance between individuals from different areas, by using multivariate methods to describe the association between explanatory (i.e., environmental variables) and response (i.e., genomic distances) variables (summarized in Riginos et al., 2016). Both population genomics and spatial ecology have their limitations, but combining these approaches allows these potential weaknesses to be better addressed and reinforces the interpretation of the observed marine connectivity scheme. Furthermore, coupling population genomics and spatial ecology approaches with information about the demographic history of species and populations can deliver a broader portrait by delineating the moment and the geographic conditions (i.e., allopatry vs sympatry) under which the genetic clusters identified in the population genomics approach formed.

Genetic clusters can be formed following habitat fragmentation and climatic/environmental change, for instance as it occurred during the Quaternary period (Hewitt, 2000; Rougeux et al., 2017). This period characterized by change in important environmental driver has shaped the connectivity of populations and the dynamic distribution of marine species in the North Atlantic (Maggs et al., 2008). Demographic modeling is fundamental toward determining whether species diverge in the face of gene flow under the action of divergent ecological selection alone (i.e., ecological speciation, Rundle and Nosil, 2005) or if an initial isolation period precedes subsequent divergence (Endler, 1977; Roux et al., 2016). Methods, either using coalescent simulations (Roux et al., 2014; Roux et al., 2016) or solving the site frequency spectrum through a diffusion approach (Gutenkunst et al., 2009), are suitable to test for these scenarios and also to model local effects in the genome, which can improve demographic inferences (Cruickshank and Hahn, 2014; Ewing and Jensen, 2016; Rougeux et al., 2017; Roux et al., 2014; Schrider et al., 2016; more details provided in the methods).

With more than 110 closely related species worldwide (Hyde and Vetter, 2007), the marine genus *Sebastes* has attracted the attention of evolutionary biologists that aim to study their speciation history (Johns and Avise, 1998). Redfish are ovoviviparous (i.e., internal fertilization), resulting in lecithotrophic larvae feeding exclusively on the yolk of the egg (Johns and Avise, 1998). In the North Atlantic Ocean, *Sebastes mentella* Travin 1951 and *S. fasciatus* Storer 1954 are two well‐suited species to study speciation that have overlapping (i.e., sympatric) and nonoverlapping (i.e., allopatric) distributions. Both species co‐occur in the Northwest Atlantic and inhabit at different depth intervals (Atkinson, 1987). The barrier to gene flow among *Sebastes* spp. in the North Atlantic Ocean is partially porous. In the Gulf of St. Lawrence (GSL) where S. *mentella* and S. *fasciatus* live in sympatry, *S. mentella* releases its larvae approximately three to four weeks earlier than *S. fasciatus* (Sévigny et al., 2000). Yet, evidence of introgression between *S. mentella* and *S. fasciatus* is present across their overlapping distribution but also between *S. mentella* and *S. norvegicus* or *S. viviparous* in the Northeast Arctic (Roques et al., 2002; Saha et al., 2017). Both *S. mentella* and *S. fasciatus* also show some intraspecific population structure.

For *S. mentella*, two ecotypes have been characterized across the North Atlantic Ocean, namely *S. mentella* shallow and *S. mentella* deep, which inhabit specific depths between 300 and 500 m and greater than 500 m, respectively (Cadrin et al., 2010; Saha et al., 2017; Valentin et al., 2014). Environmental gradients related to depth were suggested to play a role in the diversification of *S. mentella* ecotypes (Shum et al., 2014). *S. fasciatus* inhabits depths between 100 and 400 m and was previously characterized with weak population structure in the Northwest Atlantic using 13 microsatellite loci (Valentin et al., 2014). Along the Canadian coast, four populations were possibly present, namely (1) Gulf of Maine, (2) Gulf of St. Lawrence—Laurentian Channel, (3) Bonne Bay, and (4) Grand Banks—northward areas.

**FIGURE 1:**
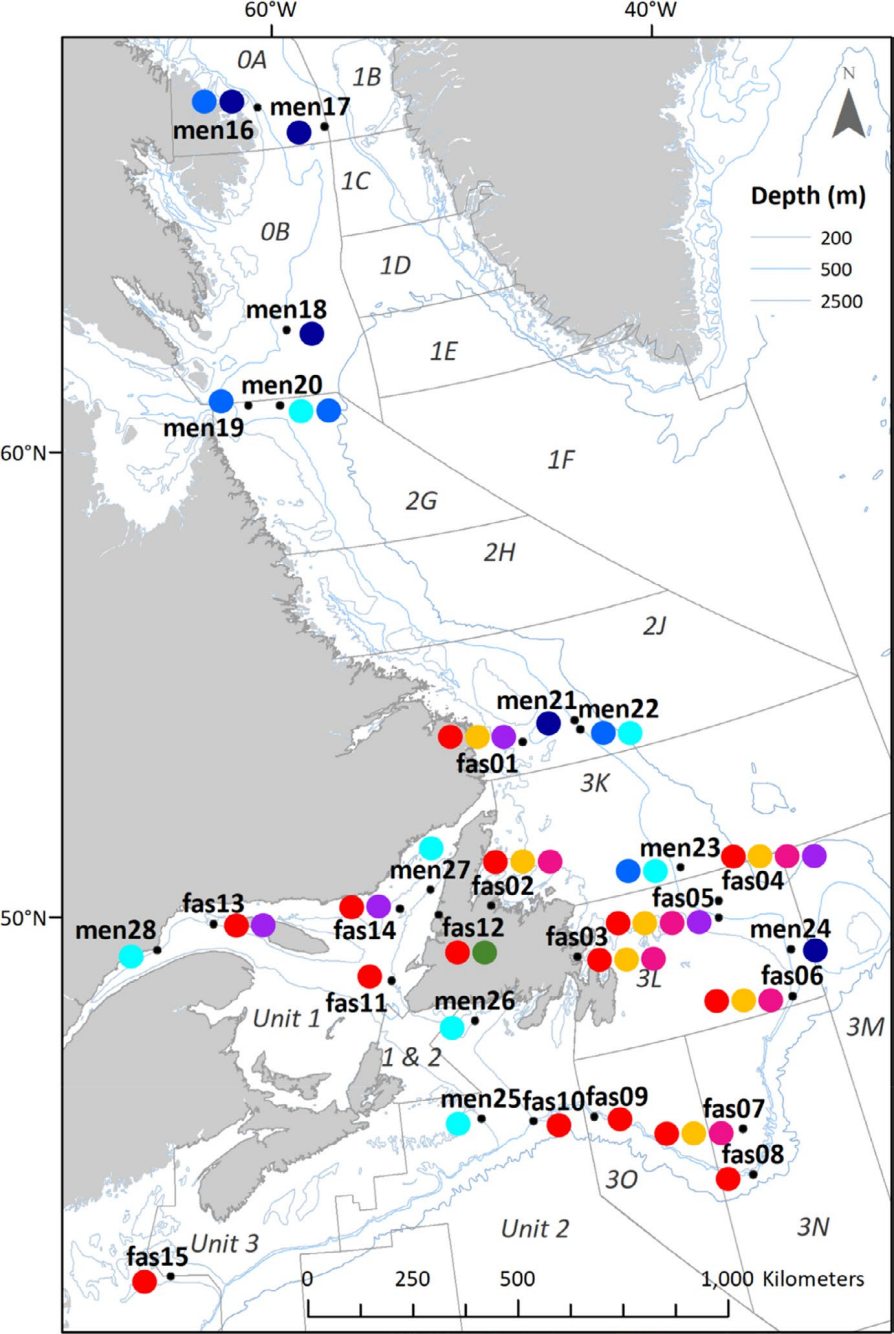
Map of the 28 sampling sites of *Sebastes mentella* and *S. fasciatus* along the Northwest Atlantic. Delineation of the current management units specific to redfish species (i.e., Units 1 to 3) and NAFO divisions are presented. Each sampling site is represented by a black point, and adjacent colored circles represent genetic clusters detected at this site. A genetic cluster was indicated as detected when at least an individual shows at least 50% of ancestry to this genetic cluster. There are three ecotypes described in *S. mentella*: GSL (cyan), shallow (blue), and deep (dark blue). The unknown *S. mentella* genetic cluster is identified in light gray. There are five populations observed in *S. fasciatus*: *I* (purple*)*, *II* (orange), *III* (red), *IV* (green), and *IV* (pink)

Redfish populations in the Northwest Atlantic have been extensively fished since the late 1950, with the fishery initially managed as three stocks established by NAFO (Northwest Atlantic Fisheries Organization). In 1993, this management plan was redefined based on a stronger biological basis, resulting in six Canadian management units (i.e., Unit 1, Unit 2, Unit 3, and NAFO divisions 2 + 3K, 3LN, and 3O, see Figure 1). A rapid decrease in landings since 1993 led to a moratorium on fishing in Unit 1 starting in 1995. In 2010, the Committee on the Status of Endangered Wildlife in Canada (COSEWIC) designated Unit 1 *S. mentella* as endangered and *S. fasciatus* as threatened. Recently, however, the abundance of *Sebastes* spp. juveniles located in the northern GSL was reported to be 230 times and 4 times higher in 2017 than their respective average abundances for the period 1993‒2012 (Senay et al., 2019). The stronger recruitment levels indicating new cohorts in the GSL may be a precursor to stock recovery and present hope for a potential future re‐opening of the redfish fishery.

While population genetic structure in *S. mentella* and *S. fasciatus* has been intensively investigated using microsatellites, no study has yet used a genome‐wide dataset to assess population genomic structure, spatial ecology, and demographic history to infer how contemporaneous and historical three‐dimensional geographic drivers played a role in the formation of distinct genetic clusters, at the species, ecotype, and population levels. Here, our aim was to genotype *S. mentella* and *S. fasciatus* individuals sampled at different depths using genotype by sequencing (GBS) to (i) delineate the contemporary population genomic structure in *Sebastes* spp. located in the Northwest Atlantic Ocean, including the recent GSL cohorts observed, (ii) assess how space, fishing depth, and sampling years explain the contemporary distribution of species, ecotypes, and populations, and (iii) characterize the demographic history of speciation.

## 2 METHODS

### 2.1 Sampling and molecular techniques

From 2001 to 2015, we collected a total of 860 *Sebastes* spp. individuals, captured from 28 sampling sites in the Northwest Atlantic Ocean (Table 1 and Figure 1). Geographic coordinates of sampling sites are average latitude and longitude of sampling locations of all individuals considered in each site (see Table S1 for sampling location of each individual). Each site was sampled in only one year. For most sampling sites, adults were sampled except in the GSL and adjacent areas where juveniles of the new cohort were sampled (Figure S1). Individuals used for genotyping were selected based on their geometric morphometrics or their population classification using the 13 microsatellites developed by Valentin et al. (2014). Only individuals that were accurately identified at the species level and *S. mentella* ecotype level (i.e., shallow and deep only) were included in this study (Table 1 for detailed information). This sampling strategy consequently reduced the proportion of admixed individuals between the species and the ecotype in this study. This sampling bias is considered in the interpretation of the results. Genomic DNA was extracted using the DNA tissue kit protocol from Omega Bio‐tek. DNA quality and quantity were checked on 1% agarose gels. Libraries were prepared based on a GBS protocol modified from Mascher et al. (2013) using *Pst*I and *Msp*I (details in Perreault‐Payette et al., 2017). Individuals were barcoded with unique sequences and pooled in multiplexes of 48 individuals per library. Libraries were then sequenced on Ion Torrent Proton P1v2 chips.

**TABLE 1:** Sampling sites analyzed in this study with year sampled, latitude, and longitude information as well as sampling depth

Code	Year	Longitude	Latitude	Ngen	Depth	Genetic group
fas01	2014	−54.07	52.93	29	387	Fasciatus
fas02	2015	−56.17	49.58	34	NA	Fasciatus
fas03	2015	−53.72	48.16	35	NA	Fasciatus
fas04	2014	−48.86	48.6	28	663	Fasciatus
fas05	2014	−49.01	48.25	35	383	Fasciatus
fas06	2014	−47.47	46.2	35	292	Fasciatus
fas07	2015	−50	43.76	30	253	Fasciatus
fas08	2015	−50.05	42.76	19	629	Fasciatus
fas09	2015	−54.2	44.73	23	306	Fasciatus
fas10	2002	−56.03	44.87	32	448	Fasciatus
fas11	2002	−59.74	48.3	32	248	Fasciatus
fas12	2002	−57.95	49.57	30	NA	Fasciatus
fas13	2013	−65.41	49.84	25	228	Fasciatus
fas14	2001	−59.18	49.82	30	217	Fasciatus
fas15	2001	−67.07	42.32	27	297	Fasciatus
men16	2008	−61.16	67.14	48	458	Shallow
men17	2008	−57.78	66.58	30	586	Deep
men18	2015	60.88	62.44	29	547	Shallow
men19	2015	−62.9	60.91	22	288	Shallow
men20	2015	61.54	60.86	25	319	Shallow
men21	2014	−52.09	53.14	34	606	Deep
men22	2014	−51.96	52.93	35	455	Shallow
men23	2014	−49.78	49.51	34	311	Shallow
men24	2014	−47.09	47.17	36	431	Deep
men25	2014	−57.56	45.11	30	290	Gulf
men26	2014	−57.3	47.2	29	234	Gulf
men27	2013	−58.08	50.13	31	228	Gulf
men28	2013	−67.3	49.31	33	230	Gulf

Note

We used a code associated with the species identified (fas or men) and selected at the sampling location (01 to 28) to simplify graphic representations. We indicated the number of individuals genotyped per sampling site (Ngen) and the genetic group (Genetic group) inferred by Valentin et al. (2014) using 13 microsatellites.

### 2.2 Bioinformatics and genotyping

We used the *process radtags* module available in STACKS v.1.46 to demultiplex libraries (Catchen et al., 2013). Reads were truncated to 80 bp, and adapter sequences were removed with CUTADAPT (Martin, 2014). Then, reads were aligned to the reference genome of *Sebastes aleutianus* (id GCA_001910805.1_ASM191080v1) using BWA‐MEM (Burrows–Wheeler Aligner‐Maximal Exact Match) algorithm with default parameters (Li, 2013). Loci were required to have a minimum stack depth of three (m = 3) among reads with potentially variable sequences and identified using *pstacks*. The catalogue of consensus loci was created allowing up to three mismatches per sample. Genotypes were obtained after running *populations* module of STACKS v.1.46. SNPs were kept according to the following consecutive filtering steps and guidelines indicated in Benestan, Ferchaud, et al. (2016). First, SNPs genotyped in at least 60% of the individuals were retained. Potential paralogs were excluded by removing markers showing heterozygosity > 0.60 and −0.50 < F_IS_ < 0.50 within sites, in more than 60% of the sites. Only SNPs showing a global minor allele frequency (MAF) > 0.01 or at least a MAF > 0.05 in one location were retained for the analysis. Only one SNP was kept per locus in order to avoid bias due to linkage disequilibrium (Table S2). This dataset was used for the analyses of population structure, the index of genetic differentiation *F*_ST_, the assignment tests, and the investigation of multivariate analyses. Another dataset of SNPs without the use of MAF filtering was also generated, with a total of 112,926 SNPs, to use with demographic models (more details in the next section). The resulting filtered VCF files were converted into the file formats necessary for the following analyses using PGDSpider v.2.0.5.0 (Lischer and Excoffier, 2012).

### 2.3 Population structure and validation with assignment tests

Clustering among samples was inferred using the Bayesian clustering method implemented in the program ADMIXTURE v.1.23 (Alexander and Lange, 2011). Admixture analyses were run using 20,000 bootstraps, and the number of clusters was set from 1 to 29 (*K*) (n + 1 sampling sites). The support for different values of *K* was assessed according to the likelihood distribution (i.e., lowest cross‐validation error) as well as a visual inspection of the co‐ancestry values for each individual. Admixture analyses were also performed at the species level in order to investigate finer patterns of genomic structure. A PCA, implemented in the *adegenet* package (Jombart, 2008), was applied to the 834 individuals genotyped at both the 13 microsatellites (dataset from Valentin et al., 2014) and at the filtered 24,603 SNPs (see results), in order to compare the degree of resolution obtained using these two types of genetic markers. The first two principal components were visualized using *ggplot2* package available in R (Wickham, 2011). To determine the extent of genetic differentiation among the 28 sampling sites, we estimated pairwise Weir and Cokhram *F*_ST_ using the *fct_84* function available in the *assigner* package (Gosselin et al., 2020), using all 24,603 SNPs.

Two runs of assignments tests were performed to test for (1) the validity of population structure and (2) the number of SNPs necessary for accurate assignment. The first assignment test aimed to assess the validity of microsatellite classification by using genetic clusters inferred for each individual using ADMIXTURE. This first run was performed in GENODIVE (Meirmans and Van Tienderen, 2004) with the frequentist method of Paetkau et al. (2004) using 1,000 permutations. For the second run, our objective was to identify the number of SNP markers required to perform an efficient and accurate assignment test at the species and ecotype levels. For this purpose, we considered a subset of “pure” individuals for ecotypes and populations that had a q‐value higher than 0.9 to a genetic cluster according to species‐specific ADMIXTURE results. Then, we tested the assignment test success relatively to 1, 2, 10, 100, 200, 300, 400, 500, 600, 700, 800, 900, and 1000, and all the polymorphic markers were genotyped (for this subset of individuals, 24,219 polymorphic markers). The second run was achieved using the function *assignment_ngs* available in the package assigner (Gosselin et al., 2020) with the following parameters: *sampling method = “ranked,” thl = 0.3, iteration_subsample = 10,* and *assignment analysis = “gsi_sim*.*”* This function was designed to simultaneously test how many genetic markers were needed to accurately assign individuals at a species and population level while correcting for the problem of high‐grading bias (Anderson, 2010).

### 2.4 Drivers explaining genomic distances

We conducted distance‐based redundancy analyses (db‐RDA; Legendre and Andersson, 1999) to investigate the variables explaining redfish genomic variation among and within species. RDA is a direct extension of multiple linear regressions to model multivariate response data (Legendre and Gallagher, 2001). First, we calculated individual Euclidean genomic distance among individuals using the *dist* function available in package *adegenet* in R as described on the https://popgen.nescent.org webpage (Kamvar et al., 2017). The individual Euclidean genomic distance matrix was then used as response variable, while the explanatory variables included spatial patterns through Moran’s eigenvector maps (MEMs, Borcard and Legendre, 2002), fishing depth, and sampling years. We performed a principal coordinate analysis (PCoA) on the individual Euclidean distances, and we kept the resulting principal component (PC) axis. These values represent the position of individuals in the genomic multivariate space, and they can be used as response variable in the db‐RDA. MEMs are derived from spectral graph theory and characterize a wide range of autocorrelation structures based on the survey design (i.e., distances between the *n* sampling locations; Dray et al., 2006). Therefore, it is a spectral decomposition of the spatial relationships among the 59 GPS points of our entire dataset. This decomposition generates (*n* ‐ 1) eigenfunctions, which are orthogonal variables that can be used in statistical models as explanatory variables representing spatial structure at different scales among the studied locations. Given the large spatial extent of the study area, distances among locations accounted for the earth curvature using the *gcd.hf* function in the *codep* package. To compute the MEMs, the truncation threshold was set to the length of the longest edge of the minimum spanning tree and only positive MEMs were retained using the *dbmem* function in the *adespatial* package. At three sampling sites (i.e., *fas02*, *fas03*, and *fas12*), depth was not recorded and therefore the median of depth distribution was used instead. Years were coded as dummy variables and treated as factors, therefore not assuming a linear relationship among years, but testing for annual differences in redfish genomic variation. To overcome multicollinearity among variables, we used the variance inflation factor with a threshold of 5, meaning that if a strong correlation was found between two variables, we retained only one of the two variables.

A global db‐RDA model was run with all explanatory variables, and model probability and adjusted coefficient of determination (adjusted *R^2^*) were calculated. The adjusted *R^2^* was quantified using the *RsquareAdj* function in *vegan* and accounts for the number of observations and number of degrees of freedom in the fitted model (Legendre et al., 2011; Peres‐Neto et al., 2006). A selection of variables contributing to the explained variation was achieved by using both forward and backward selection, and a stopping criterion (*ordiR2step* function in the *vegan* package, Blanchet et al., 2008). This criterion limits overfitting by preventing selected variables to explain more variation than the full model developed with all explanatory variables. The same approach was performed within species, using a genomic distance matrix computed for *S. mentella* and *S. fasciatus* separately, and subsets of explanatory variables associated with these individuals. Biplots were produced with a scaling of type 1 to illustrate distances among objects (i.e., individuals) and relationships with selected environmental variables. Species, ecotypes, and populations were identified by different colors in biplots to illustrate how genetic clusters were associated with selected variables. Maps of MEMs associated with redfish genomic variation were presented to visualize the most significant spatial scales.

### 2.5 Demographic history of genomic divergence

We used the diffusion approximation framework implemented in *dadi* and modified by Tine et al. (2014) to compare the joint site frequency spectrum (JSFS) of our data to those simulated under different demographic models (Gutenkunst et al., 2009). These models incorporate the effects of selection at linked sites locally affecting population size (*Ne)* and the effect of differential introgression affecting the rate of migration (*m*) along the genome (Rougeux et al., 2017; Tine et al., 2014). During the divergence process, accumulation of barriers to gene flow tends to reduce the effective migration rate locally in the genome (Barton and Bengtsson, 1986), while selection at linked sites increases the effect of genetic drift through local reduction in effective population size (Charlesworth et al., 1993). Neglecting these effects can bias demographic inferences (Ewing and Jensen, 2016; Schrider et al., 2016), but modeling different local migration rates *m* and *Ne* along the genome can help overcome this bias.

We considered four alternative models of historical divergence namely (i) a model of strict isolation (SI), (ii) a model of divergence with continuous gene flow or isolation with migration (IM), (iii) a model of divergence with migration during the first generation of divergence or ancient migration (AM), and (iv) a model of secondary contact (SC). Under IM, the two diverging populations exchange migrants at a constant rate at each generation and this gene flow can be asymmetric, so that two independent migration rates *m_12_* (from population 2 to population 1) and *m_21_* (from population 1 to population 2) were modeled. Under the SC model, the population evolved in strict isolation between *T_split_* (i.e., time of the split of the ancestral population) and until *T_sc_* (i.e., time of secondary contact) where a secondary contact (gene flow) occurs continuously up to present time. Gene flow was modeled as *M = 2Nref.m*. Heterogeneous introgression was modeled using two categories of loci occurring in proportions *P* (i.e., loci with a migration rate *M_12_* and *M_21_*) and *1‐P* (i.e., loci with a reduced effective migration rate *Me_12_* and *Me_21_*) across the genome. The same procedure was used to account for linked selection by defining two categories of loci with varying effective size. Then, we quantified linked selection using a Hill–Robertson scaling factor (*Hrf*, Rougeux et al., 2017) in order to relate the effective population size of loci influenced by linked selection (*Nr = Hrf* * *Ne*) to that of neutral loci (*Ne*). Three JSFS were constructed using 112,926 SNPs available in the raw dataset: (i) *S. fasciatus* and *S. mentella* shallow, (ii) *S. fasciatus* and *S. mentella* deep, and (iii) *S. mentella* shallow and *S. mentella* deep. It is important to note that *S. mentella* GSL and *S. fasciatus* populations were not included since the complexity of the introgression pattern observed for those populations would make difficult the interpretation of demographic models. Details of the filtering procedure are provided in supplementary materials. Model choice was performed using Akaike’s information criterion (AIC) and ΔAIC values under each given model. We used a generation time of 9.05 years for species comparisons and of 9.8 years for ecotype comparisons (Gascon 2003) and a mutation rate of 1e‐8 mutation/bp/generation (e.g., Rougeux et al., 2017; Tine et al., 2014) for parameter estimation. Standard deviations around parameter estimates were obtained using the Godambe information matrix (Coffman et al., 2016) with 100 bootstraps. Finally, in order to place the population along the divergence continuum proposed by Roux et al. (2016), we computed the net divergence (Da) using haplotypes vcf file available from STACKS. The *mscalc* pipeline (Roux et al., 2013) was used to perform these computations after filtering the dataset by removing missing data and nonpolymorphic site as above.

## 3 RESULTS

### 3.1 Two species with intraspecific geographic structure

**FIGURE 2:**
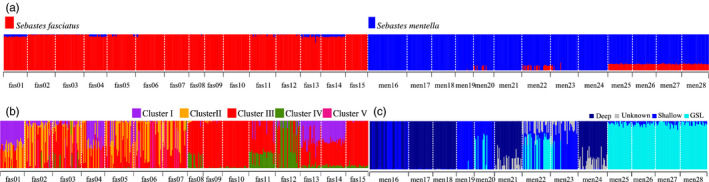
Population genomics analyses for inter‐ and intraspecific structure. Each individual is represented by a thin vertical line, which is partitioned into colored segments that represent the individual’s estimated ancestry into one of the genetic clusters observed using 24,603 SNPs. ADMIXTURE plots for (a) K = 2 for both species (n = 860), (b) K = 5 for *S. fasciatus* (n = 444), and (c) K = 4 for *Sebastes mentella* (n = 416). There are three ecotypes described in *S. mentella*: GSL (cyan), shallow (blue), and deep (dark blue). The unknown *S. mentella* genetic cluster is identified in light gray. There are five populations observed in *S. fasciatus*: *I* (purple*)*, *II* (orange), *III* (red), *IV* (green), and *IV* (pink)

Following the filtering steps shown in Table S2, we genotyped 24,603 SNPs (mean genotyping rate = 95%) in 860 individuals: 416 and 444 individuals in *S. mentella* and *S. fasciatus*, respectively. ADMIXTURE analysis revealed two highly differentiated genetic clusters (Figure 2a), with the cross‐validation (CV) error that dropped from 0.382 for K = 1 to 0.225 for K = 2. Examination of K = 2 confirmed the existence of the two species with individuals showing always more than 90% of ancestry to their own genetic cluster, except for the GSL where admixture between *S. mentella* and *S. fasciatus* was observed. Indeed, all *S. mentella* individuals of the sampling sites from the GSL to the Laurentian Fan (i.e., *men25*, *men26*, *men27*, and *men28*) consistently showed an average of 18% of *S. fasciatus* ancestry (Figure 2a). Some *S. mentella* individuals located from 3K to 2G NAFO divisions also showed ancestry with *S. fasciatus* (between 2.1 and 8.6%, Figure 2a). Inversely, we observed an average of 6% *S. mentella* ancestry within *S. fasciatus* samples from offshore East Newfoundland and North GSL (i.e., *fas01*, *fas04*, *fas13*, and *fas14*, Figure 2a). From this K = 2, the CV progressively decreased until being the smallest at K = 8 (CV = 0.209; Figure S2) where a putative population structure within each species tended to appear and was similar to the one highlighted when each species dataset was run separately (Figure 2b,c).

For *S. mentella*, the lowest cross‐validation value was for K = 4 (CV = 0.222). The four genetic clusters corresponded to two ecotypes already documented, shallow and deep, a new ecotype, GSL, and a fourth cluster designated as “Unknown” that had an unclear spatial distribution, with no individual having more than 50% ancestry of that cluster (Figure 2c). The Unknown cluster is not considered as an ecotype since its spatial distribution does not make the link with a particular habitat. As expected, *S. mentella* deep and shallow ecotypes were observed along the continental edge. The GSL ecotype was the unique ecotype observed among the juveniles of the 2011 cohort in the GSL (Figure 1). *S. mentella* ecotypes had distinct distributions in the Northwest Atlantic, albeit partially overlapping (Figure 1). For instance, *S. mentella* deep and shallow ecotypes were both sampled in *men16*. Also, 33 individuals sampled outside the GSL, in *S. mentella* shallow sampling sites (i.e., 7 individuals in *men20,* 22 individuals in *men22,* and 4 individuals in *men23*), were inferred to belong to GSL ecotype, suggesting the presence of a mixed ecotype composition in the NAFO divisions from 2G to 3K (Figures 1 and S3). Indeed, these individuals inferred to belong to GSL ecotype on the Labrador Shelf were adults (while only immature individuals were sampled in the GSL; Figure S1) and had a maximum estimated ancestry of 65% with GSL ecotype (Figure 2c). The presence of this mixed ecotypes composition on the Labrador Shelf was detected with SNP markers, whereas overlap between ecotypes was observed with the microsatellites (Figure S4).

For *S. fasciatus,* the genetic clusters detected were less obvious than for *S. mentella* since CV values dropped around K = 5 (CV = 0.196) and K = 8 (CV = 0.195). Yet, the clustering observed at K = 8 was less clear that the one documented at K = 5 since three genetic clusters out of 8 chaotically gather a few numbers of individuals (< 6 individuals) per sampling locations totalizing less than 18 individuals per cluster. In K = 5, 60% of all the *S. fasciatus* individuals (i.e., 266 out of 444 individuals) showed ancestry> 50.0% with population *III* (red; Figure 2b). The population *III* was present in all sampling sites, except *fas01* (Figure 1), and was more frequent (>90.0% of individuals) in southern distribution of *S. fasciatus*, namely *fas09, fas10,* and *fas15*. The other four populations had different geographic distributions (Figure 1): population *I* (purple; *fas01, fas04, fas05, fas13,* and *fas14;* n = 50), population *II* (orange; *fas02* to *fas07*; n = 81), population *IV* (green; *fas12*; n = 17), and population *V* (pink; *fas01, fas04, fas05, fas06, fas11, fas13,* and *fas14*; n = 30).

We compared *F*_ST_ values and the results of ADMIXTURE analyses at the global and genetic cluster scales in order to examine the extent of inter‐ and intraspecific genetic differentiation. The heatmap based on *F*_ST_ values gave similar results to the ADMIXTURE analyses. Both analyses identified the two species, as well as the three ecotypes and five populations within *S. mentella* and *S. fasciatus,* respectively (Figure S5). The smallest *F*_ST_ value was between *men27* and *men28* (*F*_ST_ = 0.0008, CI_low_ = 0.0004, CI_high_ = 0.0013), whereas the largest *F*_ST_ value was between *fas12* and *men18* (*F*_ST_ = 0.6086; CI_low_ = 0.6025, CI_high_ = 0.6147; Figure S5). We observed a pronounced genetic differentiation between samples from the two species (*F*_ST_ = 0.5050, P‐value < 0.001), which was in line with ADMIXTURE results showing that the delimitation of the two species captured the majority of the signal of genetic differentiation. Within *S. mentella*, *F*_ST_ values were twice larger—on average 0.0545 (ranging from 0.0004 to 0.1124)—than within *S. fasciatus—*on average 0.0257 (ranging from 0.0019 to 0.0715), which again was similar to ADMIXTURE finding a clearer population genomic structure in *S. mentella* than in *S. fasciatus*. Furthermore, the *F*_ST_ heatmap showed that the sampling location *men22* is genetically more similar to GSL than to shallow ecotype (Figure S5), which is likely due to a mixed ecotype composition of GSL and shallow individuals at the *men22* sampling site as indicated by the ADMIXTURE analysis (Figure 2c).

### 3.2 Accuracy of assignment test at the species and ecotype level

We tested the validity of the clustering found by ADMIXTURE between and within species by performing individual assignment tests using all 860 individuals from both species. When considering the species and ecotype identity (shallow or deep) inferred from microsatellites and using the new 24,603 SNP markers genotyped, we obtained 100% assignment success at the species level.

**FIGURE 3:**
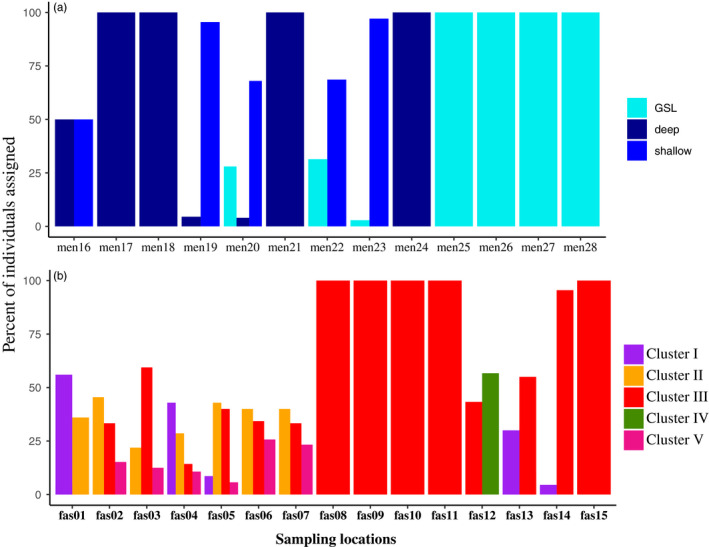
Assignment tests. Bar graph showing the percent of individuals correctly assigned for each sampling site and considering the three ecotypes and the five populations inferred from ADMIXTURE analyses in (a) *Sebastes mentella* and (b) *S. fasciatus*. Note that assignment tests were run by considering the genetic group inferred for each individual using the species‐specific ADMIXTURE analysis

At the ecotype level for *S. mentella*, we assigned 100% of the individuals coming from GSL and deep ecotypes, while for shallow ecotype assignment, success varies from 68% to 97% (except for *men16* where deep and shallow ecotypes were both sampled). The sampling locations *men22* and *men20* showed the highest percent of misassigned individuals—on average 32%—and these individuals were inferred to belong to *S. mentella* GSL, which corroborate the pattern seen in ADMIXTURE (Figure 2c) and *F*_ST_ analyses (Figure S5). Only a few percent of misassigned individuals sampled in shallow ecotype locations (i.e., *men19*, *men20*, *men22*, and *men23*) were assigned to deep ecotype (Figure 3a).

The assignment success within *S. fasciatus* considering ADMIXTURE results revealed a very high assignment success always superior to 96.5% for every population. The populations *II* and *V* exhibited the lowest, albeit high, assignment success (96.5% and 97.5%, respectively), while populations *I*, *III*, and *IV* reached 100% of assignment success. Despite this high assignment success at the population level, we observed that sampling locations were actually composed of different populations except for *fas08*, *fas09*, *fas09, fas11, and fas15* where all individuals belong to population *III* (Figure 3b). The only population that matched a unique sampling site (*fas12*) was population *IV* (Figure 3b).

**FIGURE 4:**
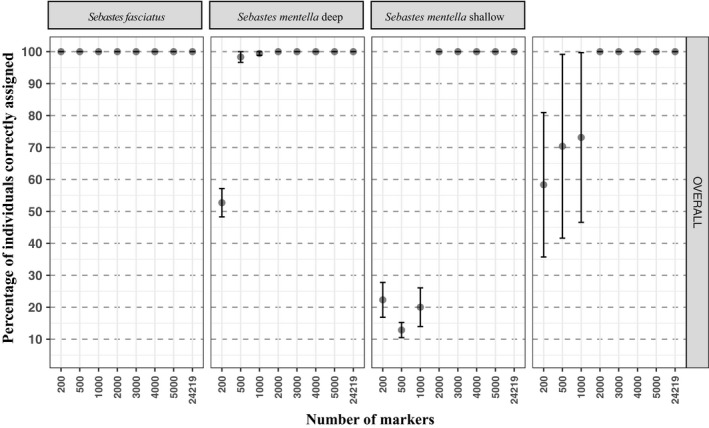
Assignment success related to the number of markers used (from 1 to 24,219 SNPs) based on ranked *F_ST_* values of SNPs and using THL (Training, Holdout, Leave‐one‐out) method in *S. mentella* GSL, *S. mentella* shallow, *S. mentella* deep, and *Sebastes fasciatus*

By selecting a subset of 258 “pure” individuals assigned with up to 90% of ancestry to their genetic cluster, using ADMIXTURE results, we then assess the number of SNP markers required to reach a high assignment success at the species and ecotype levels (i.e., > 90%). This analysis revealed that only two SNPs (Figure S6) were needed to reach an assignment success of 100% at the species level while the use of a minimum of 2,000 highly divergent markers was required at the ecotype level for *S. mentella* (Figures 4 and S6).

### 3.3 Inter‐ and intraspecific genomic distances explained by spatial distribution, fishing depth, and sampling years

As the geographic distributions of *Sebastes* species, ecotypes, and populations surveyed in this study seemed distinct but overlapping (Figure 1), we investigated how environmental variables could explain the genomic distance using a db‐RDA (see Methods for more details). We used the individual sampling characteristics (i.e., geographic coordinates, depth, and year) for these analyses. For all samples, depth ranged from 134 to 708 m, with samples from *S. mentella* and *S. fasciatus* ranging between 228 to 708 m and 134 to 678 m, respectively. There are a total of 6 sampling years for both species (i.e., 2008, 2013, 2014, and 2015 for *S. mentella*; 2001, 2002, 2013, 2014, and 2015 for *S. fasciatus*).

**FIGURE 5:**
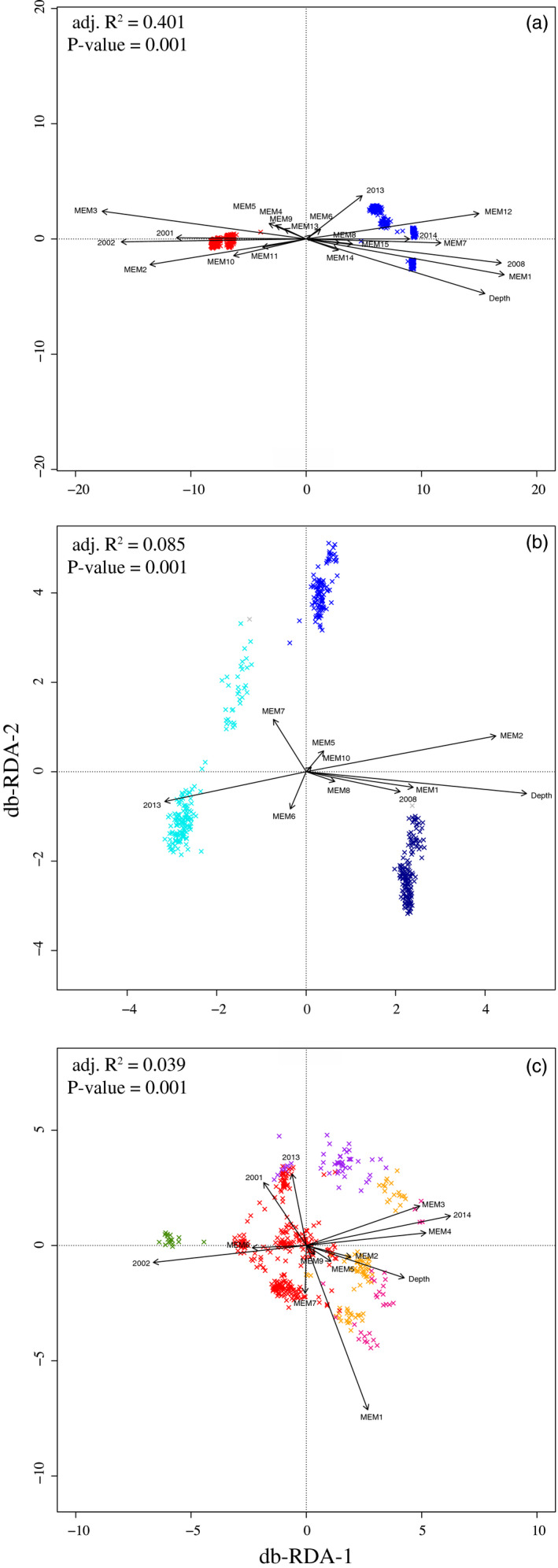
Db‐RDA ordination biplot (scaling 1), which preserves distances among objects and environmental variables for (a) 860 individuals, (b) 416 *S. mentella* individuals, and (c) 444 *S. fasciatus* individuals. (a) *S. mentella* is shown in blue and *S. fasciatus* in red. (b) There are three ecotypes described in *S. mentella*: GSL (cyan), shallow (blue), and deep (dark blue). The unknown *S. mentella* genetic cluster is identified in light gray. (c) There are five populations observed in *S. fasciatus*: *I* (purple*)*, *II* (orange), *III* (red), *IV* (green), and *IV* (pink)

First, explanatory variables were selected for the complete dataset (i.e., including both species). The elaboration of the MEMs based on geographic coordinates created 19 spatial explanatory variables (MEM1 to MEM19). The first explanatory variables (e.g., MEM1) corresponded to large spatial scales, while the last ones (e.g., MEM19) corresponded to fine spatial scales. A total of 21 explanatory variables were retained, namely 16 spatial variables, fishing depth, and sampling years (Table 2 and Figure 5a). The response variable was derived from a PCoA on the genetic distance matrix including 860 individuals from both species resulting in 859 PCs (i.e., *n*‐1) representing individuals’ genomic variation. All 859 PCs were used as response variables in the db‐RDA.

**TABLE 2:** List of selected explanatory variables contributing to the genomic variation present among 860 individuals from both species

Variable	Cum Adjusted *R* ^2^	AIC	*F*	*p*‐value
MEM3	0.07597	7897.0	71.6277	.002
MEM1	0.14805	7828.2	73.5881	.002
MEM12	0.20215	7772.7	59.1131	.002
MEM2	0.24848	7722.3	53.7701	.002
Y2014	0.29454	7668.9	56.8162	.002
MEM7	0.33751	7615.8	56.3997	.002
MEM10	0.34889	7601.9	15.9108	.002
Y2008	0.35457	7595.4	8.4877	.002
MEM8	0.36108	7587.7	9.6702	.002
MEM15	0.36622	7581.7	7.9016	.002
Y2013	0.37161	7575.3	8.2740	.002
MEM4	0.37678	7569.2	8.0411	.002
MEM9	0.38072	7564.8	6.3821	.002
MEM14	0.38467	7560.2	6.4335	.002
MEM5	0.38793	7556.6	5.5016	.002
Depth	0.39026	7554.3	4.2297	.002
MEM11	0.39317	7551.2	5.0350	.002
Y2002	0.39526	7549.2	3.9209	.002
MEM6	0.39706	7547.6	3.5122	.002
Y2001	0.39919	7545.6	3.9744	.002
MEM13	0.40057	7544.6	2.9218	.002

Note

Variable name, cumulative adjusted *R* ^2^, Akaike’s information criterion (AIC), F statistic, and probabilities (*p*‐value) are indicated.

The global db‐RDA using all explanatory variables was significant (*p*‐value = .001) and accounted for 40.1% of the genomic variation (adjusted *R* ^2^ = 0.401). Using a forward and a backward selection approach, we identified the variables explaining most of the genomic variation. The db‐RDA biplot shows distances among objects and relationships with selected variables (Figure 5a). The first db‐RDA axis accounted for 92.3% of the explained variation, while the second accounted for 2.5%. On the left side of the biplot, *S. fasciatus*, in red, was mostly explained by the years 2002 and 2001, when most samples of that species were collected. On the right side of the figure, *S. mentella*, in blue, was mainly driven by 2008 and by greater fishing depths. MEM3 and MEM1 were the spatial variables best explaining species distribution (Figure S7).

With a subset of locations where *S. mentella* was present, 10 MEMs were obtained. The global db‐RDA for *S. mentella* using all explanatory variables (10 MEMs, fishing depth, and sampling years) was significant (*p*‐value = .001) and accounted for 8.5% of the genomic variation (adjusted *R* ^2^ = 0. 085). Variable selection identified seven MEMs, depth, and two sampling years (2008, 2013) as significant drivers of genomic variations within *S. mentella* (Table S3 and Figure 5B). The first db‐RDA axis accounted for 59.3% of the explained variation, while the second accounted for 13.7%. The first axis illustrated a gradient from the GSL ecotype in cyan on the left side, the shallow ecotype in intermediate blue in the middle, and the deep ecotype in dark blue on the right side. The second axis separated the deep ecotype in dark blue at the bottom, the GSL in cyan in the middle, and the shallow ecotype in intermediate blue at the top. Fishing depth, MEM2, and MEM1 were the variables most associated with the first axis. MEM2 corresponded to a large spatial gradient, where northern individuals shared more genetic similarities with southern individuals. MEM7 corresponded to a small spatial scale, where some individuals in the GSL or close to GSL had genomic differences compared to their surrounding counterparts (Figure S8).

Eight MEMs were obtained with the subset of locations where 444 *S. fasciatus* individuals were subsampled. The global db‐RDA (8 MEMs, fishing depth, and sampling years) was significant (*P*‐value = .001) and accounted for 3.9% of *S. fasciatus* genomic variation (adjusted *R* ^2^ = 0.039). Variable selection identified 8 MEMs, depth, and sampling years (2002, 2014, 2001, and 2013) as significant (Table S4 and Figure 5C). The first db‐RDA axis accounted for 24.2% of the explained variation, while the second accounted for 19.8%. The population *I*, shown in purple, was mostly associated with 2013 and MEM1, while the population *IV*, in green, was positively related to 2002. Populations *II, III*, and *V* in orange, red, and pink, respectively, had a large distribution in the db‐RDA space, and therefore, no clear relationships with explanatory variables could be identified (Figure 5c). MEM1 and MEM4 were the spatial variables best explaining *S. fasciatus* population distribution. MEM1 outlined a large‐scale east–west gradient. MEM4 also showed a large‐scale gradient where northeast individuals were different from their southwest counterparts (Figure S9).

### 3.4 Evidence for secondary contact at both species and ecotype level

**FIGURE 6:**
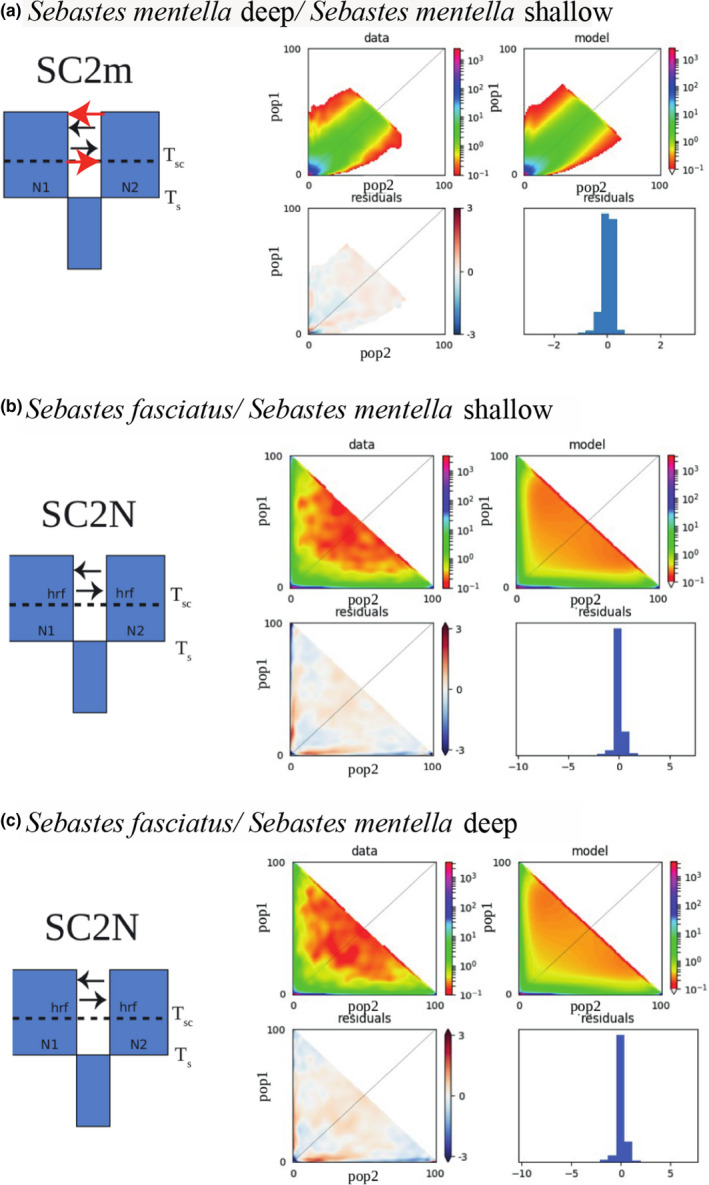
Observed best SC models (top panel) fitted to the folded site frequency spectrum (bottom panel) in the comparison between ecotypes (a) and between species (b and c). The suffix “2m” denotes heterogeneous migration and “2N” heterogeneous effective population size. AM = ancient migration. IM = isolation with migration, SC = secondary contacts, SI = strict isolation. All fittest models incorporate heterogeneous parameters, such as a heterogeneous rate of gene flow (2 m) and linked selection (2N). Each block corresponds to a population with effective population size of N1, N2, and θ. Horizontal dashed lines indicate the duration (T_sc_) of the secondary contact period after the duration (T_s_) of the allopatric period, while vertical dashed lines indicate change in effective size. Black arrows symbolize neutral gene flow, whereas red arrows symbolize heterogeneous introgression

We tested four major different demographic models, and the model choice indicated that the SC model was the best model for all three comparisons: *S. mentella* deep versus *S. fasciatus*, *S. mentella* shallow *versus S. fasciatus*, and *S. mentella* shallow *versus* deep (Table S5). In interspecific comparisons, the AIC and ΔAIC indicated that the SC model incorporating variation in drift (i.e., SC2N) received the highest support, while in the intraspecific comparisons, the model incorporating heterogeneous introgression (SC2m) was the best (Figure 6 and Table S5). Under SC2N, we found a simultaneous divergence time for the two comparisons between *S. fasciatus* and the ecotypes (i.e., *S. mentella* deep and shallow) with approximately 1 MYA (million years ago) of divergence for each comparison (see Table 3 for uncertainties around parameter estimates). The secondary contact represented on average 10.5% of the total time of divergence for these SC2N models, indicating that the species remained isolated most of their divergence time. The effective population sizes (Table 3) and the ratio of effective population sizes under all SC models revealed that both species showed large and relatively similar *Ne* (i.e., ratio *Ne*_Fas_/*Ne*_Men_ ~ 0.92). Estimates of migration rate were low (m_1_ and m_2_ < 8e‐6; Table 3), suggesting that only a few migrants have been exchanged between species since secondary contact. We further found that approximately 50% of the genome displayed a reduced population size (Q = 0.50; Table 3) due to the action of linked selection. In these regions affected by linked selection, the *Hrf* indicated that the population size was 17% of the total population size.

**TABLE 3:** Demographic parameter estimates obtained between the three major groups compared

pop1	*Sebastes fasciatus*	*Sebastes fasciatus*	*Sebastes mentella* deep
pop2	*Sebastes mentella* deep	*Sebastes mentella* shallow	*Sebastes mentella* shallow
Best model	SC2N	SC2N	SC2m
AIC	9588	9652	7426
Log likelihood	−4786	−4818	−3704
Theta	21466	2077	1908
Nref	53,000 [44,000–62,000]	51,000 [43,000–58,000]	58,000 [57,000–59,000]
Ne1	119,000 [81,000–158,000]	116,000 [88,000–143,000]	63,000 [62,000–64,000]
Ne2	127,000 [89,000–165,000]	126,000 [98,000–155,000]	89,000 [88,000–90,000]
m1 ← 2	5,3E‐06 [3,8E‐06–6,7E‐06]	6E‐06 [5,6E‐07–1,14E‐05]	4E‐04 [0–0,001]
m2 ← 1	1,7E‐06 [1,3E‐06–2,2E‐06]	3E‐06 [2,86E‐06–3,11E‐06]	2E‐04 [1e‐04–2,4E‐04]
2me1 ← 2	NA	NA	2,4E‐04 [2,2E‐04–2,6E‐04]
me2 ← 1	NA	NA	3,8E‐05 [3,3E‐05–4,4E‐05]
Tsplit	1,027,000 [912,000–1,142,000]	1,040,000 [1,024,000–1,054,000]	417,900 [416,800–419,000]
Tsc	124,700 [120,000–1290,00]	89,000 [86,000–93,000]	23,000 [22,000–24,000]
hrf	0.17	0.16	NA
P/Q	0.50	0.50	0.70

Note

AIC, Akaike’s information criterion; log likelihood, maximum likelihood; theta: effective mutation rate of the reference population, which here corresponds to the ancestral population; Nref, size of the ancestral; Ne1 and Ne2, effective population size of the compared pair; m1 ← 2 and m2 ← 1, migration from population 2 to population 1 and migration from population 1 to population 2; me12 and me21, effective migration rate estimated in the most differentiated regions of the genome; Ts, time of split of the ancestral population in the two species; Tsc, duration of the secondary contact; P, proportion of the genome freely exchanged (1‐P provides the proportion of the genome non‐neutrally exchanged); Q, proportion of the genome with a neutral Ne (1‐Q provides the proportion of the genome non‐neutrally exchanged); hrf = Hill–Robertson factor representing the reduction of Ne in the region (1‐Q) with reduced Ne.

For ecotype comparison, the median estimate of divergence time between *S. mentella* deep and shallow population was ~ 0.46 MYA under the SC2m models. The migration rate since secondary contact was higher than that observed between species and strongly asymmetric, with higher migration from the deep (*m*_12_ = 0.0003) to the shallow population (*m*_21_ = 9e‐5 Table 3). Overall, we found that ~28% (ranging from 26 to 38%) of the genome was undergoing reduced introgression since secondary contact (P parameter in Table 3). Finally, using haplotype data derived from the GBS loci, we found that the level of net divergence (Da) over all combined loci was an order of magnitude lower in intraspecific comparisons (Da ~ 0.0001) compared to interspecific comparisons (Da ~ 0.0015).

## 4 DISCUSSION

The goals of this study were to (i) assess population genomic structure with a dense marker set, (ii) investigate how environmental or temporal drivers may influence the population structure, and (iii) reconstruct the speciation history of two redfish species along the Northwest Atlantic Ocean. We confirmed the pronounced genetic distinction between *S. mentella* and *S. fasciatus*, and moderate genetic differentiation between two *S. mentella* ecotypes, deep and shallow. We identified a new ecotype of *S. mentella* GSL and at least five populations of *S. fasciatus* in the Northwest Atlantic. High individual assignment success was achieved at species, ecotype, and population levels for the first time, confirming that GBS data provide a powerful tool for traceability of the redfish fishery. Inter‐ and intraspecific genomic variations were best explained by large‐scale spatial distribution and fishing depth. We also uncovered that postglacial secondary contact was the most likely scenario to explain the speciation history of species and *S. mentella* ecotypes.

### 4.1 Highly structured *Sebastes* species in the northwest Atlantic

We observed a level of genetic differentiation spanning from weak to pronounced between the genetic clusters within both *S. mentella* and *S. fasciatus*, and all have distinct but overlapping distributions. The largest estimate of genetic differentiation was between *S. mentella* and *S. fasciatus* species (*F*_ST_ = 0.505; P‐value < 0.001), supporting that these genetic clusters form independent demographic units.

Within *S. mentella*, we confirmed the presence of deep and shallow ecotypes and identified a new ecotype, GSL in the Northwest Atlantic with more genetic similarities to shallow than deep ecotypes. It is then possible that GSL ecotype may have diverged from the shallow ecotype. The shallow and deep ecotypes were previously identified and described as distinct genetic clusters inhabiting different depths along the continental slope in past studies across the North Atlantic (Cadrin et al., 2010; Saha et al., 2017; Valentin et al., 2014). Here, we suggest to use the term ecotype to describe these ecogeographically isolated groups. Ecotype was first defined and summarized as a genetic group specific to a particular habitat (Turesson, 1922). We considered that the three genetic groups of *S. mentella* described in this study correspond to that first definition and that results obtained from the db‐RDAs have shown that the genetic distance between these ecotypes is explained by differences in habitats through space and depth. Furthermore, the genetic composition of the *S. mentella* GSL ecotype shows consistent admixture with *S. fasciatus* (i.e., 18%) and it is the unique ecotype present in the GSL, a partially isolated area.

Within *S. fasciatus*, we identified five populations occurring from the Gulf of Maine to the Labrador Shelf with highly overlapping distribution. This contrasts with the more pronounced differentiation we observed between *S. mentella* ecotypes and consequently justifies the use of the term population (instead of ecotype) to describe these genetic clusters. Nevertheless, the five populations identified are mostly associated with distinct geographic areas along the Canadian coast (Figure 1), suggesting further investigation on the population structure of this species. The population *I* was the only genetic cluster that had up to 6% of *S. mentella* ancestry, which underlined that introgression may be the main cause of the distinctiveness observed in this particular population. Moreover, a higher genetic differentiation—in line with previous microsatellite observations (Valentin et al., 2014)—was detected for the semiclosed population located in Bonne Bay Fjord (*fas12*; population *IV*), for which we hypothesize that limited water exchange or limited physical connectivity may be responsible for this pattern, as previously proposed in the red algae *Palmaria palmate* (Li et al., 2015). Still, *fas12* does not appear completely isolated and larvae may potentially migrate in the fjord since a mixture of two populations was observed at this location. Our results confirm that there is a unique population in Bonne Bay Fjord, which was in the past designated as “of special concern” because of its demographic isolation (COSEWIC, 2010); however, our results also suggest that the Bonne Bay Fjord could naturally be reseeded from other populations.

The spatially distinct distribution of species, ecotypes, and populations, as well as levels of genetic differentiation among them, suggests limited dispersal and migration of *S. mentella* and *S. fasciatus* in the Northwest Atlantic (ca. 0.505). For migratory fish species, estimates of genetic differentiation using thousands of neutral SNPs between trans‐Atlantic populations are of ca. 0.016 for Atlantic mackerel (*Scomber scombrus*; Rodríguez‐Ezpeleta et al., 2016) and ca. 0.081 for Atlantic cod (*Gadus morhua;* Berg et al., 2017). We observed on a much smaller spatial scale higher levels of genetic differentiation between species (ca. 0.5050) and within *S. mentella* (ca. 0.0545) suggesting limited dispersal during the larval stage and short migration distance. Their ovoviviparity, involving internal fertilization and larval extrusion, likely reduces the extent of dispersal and may reduce interspecific mating and produce reproductive isolation, thus favoring genomic differentiation on small geographic scales (Johns and Avise, 1998). Consequently, these genetically and ecologically distinct groups appear mostly closed (i.e., self‐recruiting) and thus need to be individually protected for proper conservation (Hellberg et al., 2002). Although hybrids have been documented between these two species in the past, no F1 was documented in a nonbiased sampling design (Roques et al., 2001). Along with our result, this tends to indicate that introgression events may be ancient between the two species, and thus, introgression is not limiting the establishment of genome‐wide barriers to gene flow between these species. Nevertheless, our current sampling design, which avoids potential hybrids by sampling only individuals clearly assignable to a priori groups, limits our interpretation of our results to properly answer to this question.

Although we identified pronounced intraspecific structure in *S. mentella*, some structure is still unexplained and more genotyping will be necessary to identify all the demographic units present in the Northwest Atlantic. Thus, an “unknown” genetic cluster was identified in *S. mentella* sampling zones (i.e., *men21*, *men22*, *men23*, and *men24*) that correspond to an area of contact with another *Sebastes* species, *Sebastes norvegicus*, which is present in the North Atlantic (Stransky, 2005). We also identified some admixture between the GSL and shallow ecotypes of the NAFO divisions from 3K to 2G. These samples were from adults and suggest that past larval migration through the Strait of Belle Isle was successful that these GSL ecotypes could reproduce with shallow ecotypes and have descendants who survived to adulthood. It would be of great interest in future studies to investigate whether the new GSL cohort has also dispersed to the Labrador Shelf and evaluate whether reproduction between GSL and shallow ecotypes can occur more than once.

It is also likely that our sampling under‐represented the *S. fasciatus* populations that may be present from the Gulf of Maine. Microsatellites indicated a clear genetic differentiation from Gulf of Maine sample (Roques et al., 2002; Valentin et al., 2014) which could not be tested with our sampling design due to the low number of individuals south of the 45^th^ parallel that were genotyped. Clearly, the total number of *S. fasciatus* individuals that were available for this study most likely reduced the power to identify populations while structure is present (Puechmaille, 2016). Indeed, the initial design of the study was the investigation of the *S. mentella* and *S. fasciatus* differentiation, with a focus on the characterization of *S. mentella* individuals in the GSL, hence the lack of *S. fasciatus* samples. Future studies should sample more *S. fasciatus* over the whole species distribution to identify an accurate number of populations.

### 4.2 Spatial distribution, depth, and other factors explaining inter‐ and intraspecific barriers to gene flow

Defining population structure in marine species remains challenging to investigate considering the absence of obvious barriers to gene flow (Palumbi, 1994). Relying on tools from spatial ecology along with population genomics, we found that spatial distribution explained most of the genetic differentiation in the redfish system while fishing depth and sampling years significantly explained from a moderate to a small proportion of the genomic distances between individuals of *Sebastes* spp. Spatial patterns were strong predictive variables for species, ecotypes, and populations, which is in concordance with the emerging results of recent seascape genomic studies (reviewed in Riginos et al., 2016) on the American lobster (*Homarus americanus*; Benestan, Quinn, et al., 2016), the sea cucumber (*Parastichopus californicus*; Xuereb et al., 2018), the red mullet (*Mullus surmuletus*; Dalongeville et al., 2018), and the Eastern oyster (*Crassostrea virginica*; Bernatchez et al., 2019). In particular, this north/south genomic structure may reflect an historical signature of a glacial refugium during the Pleistocene followed by population expansion throughout the northwest. Pleistocene glacial cycles associated with geological and climatic changes have indeed clearly played a role in shaping the contemporary distribution and abundance of marine organisms in the North Atlantic (Wares and Cunningham, 2001).

Depth may play a role in the isolation of these species and other *Sebastes* species (Ingram, 2011; Shum et al., 2015). Despite the potential influence of bathymetry on marine organisms, no recent seascape genomics studies have already investigated and evidenced how this environmental feature can drive marine population genomics. Here, we uncovered that fishing depth was a significant explanatory variable for genomic distances between species, ecotypes, and populations of *Sebastes* spp. This outcome brings new evidence that habitat depth is a strong barrier to dispersal of *Sebastes* spp and contributes to the wide literature covering the topic specifically for *S. mentella* (Cadrin et al., 2010; Stefánsson et al., 2009) and other marine species such as the deep‐sea coral (*Desmophyllum dianthus*; Miller et al., 2011), the common protobranch bivalve (*Nucula attacellana*; Jennings et al., 2013), and the small‐spotted catshark (*Scyliorhinus canicular*; Kousteni et al., 2015). We also need to highlight that while depth was the most important significant predictor of diversification within *S. mentella*, it does not explain solely the barrier to gene flow between *S. mentella* and *S. fasciatus* since both species inhabit a similar depth range in our study area. Other pre‐ or postzygotic barriers to gene flow most likely played a role in their reproductive isolation.

Another significant predictor of the overall genomic variation was the year of sampling. This result is not surprising as we sampled a specific area each year due to sampling constraints (i.e., vessel availability), and thus, this is likely due to our nonrandom sampling design. If logistically possible, future population genomic studies on *Sebastes* spp. should sample all areas at different years to accurately test whether species and populations distribution remain constant interannually.

### 4.3 Physical separation followed by secondary contacts explains the speciation history for both species and *S. mentella* shallow and deep ecotypes

Our study is the first to extensively investigate the history of speciation for the *Sebastes* species inhabiting the North Atlantic using explicit demographic simulations. Here, we discovered that scenarios of secondary contact (SC) provided the best fit to the observed data for both species and ecotypes. Furthermore, modeling the process of speciation between *S. mentella* and *S. fasciatus* has never been done before, while for *S. mentella* deep vs. shallow comparison, Saha et al. (2017) previously identified the isolation with migration (IM) model as the best model to explain the divergence observed. However, the authors did not test for secondary contact and used a limited number of microsatellite markers, which are known to quickly converge to demographic equilibrium and therefore offer limited resolution to distinguish among models of divergence with gene flow (IM) and secondary contact (SC) (Bierne et al., 2013). In contrast, SNP markers are more suited to perform demographic inferences given their lower mutation rate (Ellegren, 2000). Here, we used a much higher number of markers, thus reaching sufficient power to accurately distinguish these two models. Our parameter estimates suggested that the two species diverged first (~1 MYA) followed by a more recent split of the ecotype (~0.5 MYA). These divergence times correspond to a pronounced drop of sea level, suggesting that the redfish species may tend to become genetically isolated during periods of expansion/contraction associated with the Pleistocene glacial oscillations (2.6 million to 11,700 years ago), as previously suggested by Hyde and Vetter (2007). Under these conditions, the genetic clusters had enough time to accumulate genomic incompatibilities that could act as a postzygotic mechanism of reproductive isolation upon secondary contact. Nevertheless, these estimates should be interpreted cautiously given that deviation from the mutation rate that we used here may substantially alter demographic parameter estimates (although the ratio of estimated time of secondary contact over divergence time still applies). Overall, our results are consistent with an increasing recent report of secondary contact in a wide range of marine species such as the European anchovy (Le Moan et al., 2016), the Atlantic cod (*Gadus morhua*; Fairweather et al., 2018) and European sea bass (*Dicentrarchus labrax;* Tine et al., 2014).

Our power to infer secondary contacts between ecotypes may have been favored by strong gene flow erasing previous genetic divergence outside of barrier loci and generating an excess of variants at intermediate frequencies (Alcala and Vuilleumier, 2014). Indeed, we pointed out that ~ 30% of the genome is undergoing reduced introgression between ecotypes, in line with previous estimates for marine species—ranging from ~ 15 to 45%—suggesting that most of the genome is still largely semipermeable to gene flow (Gagnaire et al., 2018; Le Moan et al., 2016). In these conditions, it is possible that upon secondary contact with *S. fasciatus*, genetic variants under positive selection in *S. fasciatus* and under negative selection in *S. mentella* may have allowed the adaptation of the shallow ecotype to an intermediate habitat depth. The observation of GSL ecotype that displayed relatively high historical admixture with *S. fasciatus* further suggests interspecific introgression events. Testing whether introgression is adaptive (Fraïsse et al., 2014; Hedrick, 2013) and resolving its role in the subsequent colonization of different depths would require an integrative study including whole‐genome sequencing of the four *Sebastes* species (i.e., *Sebastes fasciatus*, *Sebastes mentella, Sebastes norvegicus*, and *Sebastes viviparous*) across their whole distribution range. Nevertheless, this signal of introgression between *S. mentella* and S*. fasciatus* may also be due to the fact that both species and ecotypes showed large *Ne* and recent divergence time, which may result in incomplete lineage sorting indicating that some ancestral variants are still shared. Indeed, delineating species along a continuum of genomic divergence is a major challenge in evolutionary biology but can be tackled through appropriate demographic modeling by investigating the relation between net synonymous divergence (Da) and the probability of ongoing gene flow (Roux et al., 2016). Here, we found the Da estimate was ten times higher between species than ecotypes (Da = 0.0001 *versus* Da = 0.0015), which supports the idea that (*i*) ecotypes are more recently diverged than species, as well as the fact that (*ii*) species and ecotypes fall within the “grey‐zone of speciation” (Roux et al., 2016). Overall, we highlighted that models integrating linked selection and gene flow provided the best fit to our data for both species and ecotypes. These results supported a role for linked selection in shaping differentiation landscape, as is increasingly reported in the speciation literature (Burri et al., 2015; Cruickshank and Hahn, 2014; Rougemont and Bernatchez, 2018; Roux et al., 2016; Stankowski et al., 2019).

### 4.4 Improving management of *Sebastes* spp. in the Northwest Atlantic Ocean

Despite the plethora of studies in ecology and genetics that have shed light on their limited movement and their large genetic differentiation (Roques et al., 2002; Roques et al., 2001; Valentin et al., 2014; Valentin et al., 2002), the two redfish species, *S. mentella* and *S. fasciatus*, are still often reported as “redfish”—due to their morphological similarities—without any differentiation in stock assessment, except for Unit 1 and 2 stocks where species‐specific estimates are provided (DFO, 2020), and current Canadian management plans at some management units. Yet, reproductively isolated redfish aggregations can then be at risk of locally eliminating biological diversity and overfishing with the current large‐scale management plan that does not reflect the biology of the species (Benestan, 2020). Since the collapse of the vast majority of the stocks in the Northwest Atlantic, the Canadian government has undertaken significant efforts to improve the recovery of redfish populations but more efforts may be required.

We identified unexpected asymmetric migration through the Strait of Belle Isle. The ADMIXTURE and the assignment analyses pointed out that some individuals sampled in the habitat of the shallow ecotype from the Eastern Newfoundland coast to the Hudson Strait shared some ancestry with GSL ecotype (up to 65%). Yet, on the western Newfoundland coast, in the GSL, individuals shared ancestry only with the GSL ecotype and form the large new cohorts rebuilding the stock within the GSL (Senay et al., 2019). Similarly, *S. fasciatus* individuals belonging to the cluster *V* were also identified on the Labrador Shelf and Northern Grand Banks, uncovering possible asymmetric gene flow from North GSL to Labrador Shelf. These results imply that *S. mentella* and *S. fasciatus* may disperse from the North GSL through the Strait of Belle Isle to the Labrador Shelf, as it has been reported for the snow crab (*Chionoecetes opilio;* Puebla et al., 2008) and the capelin (*Mallotus villosus*; Kenchington et al., 2015, Cayuela et al., 2019). Since adult redfish were rarely observed in the shallow Strait of Belle Isle, we propose that larval drift may play a key role in linking subpopulations. Management Unit 1 and NAFO divisions 3K and 2G tend to then exchange more than 10% of individuals, which corresponds to a maximum threshold that can be used to infer demographic independence between two areas (Hastings and Harrison, 1994; Lowe and Allendorf, 2010; Waples and Gaggiotti, 2006). As the planning for possible re‐opening of the redfish fishery in the GSL following its signs of recovery (Senay et al., 2019), the recovery of this stock may strongly influence adjacent areas to the GSL, such as the south of Labrador Shelf. We then urge for a conjoint discussion across Atlantic provinces and the south of Labrador Sea for the purpose of establishing a sustainable plan for this marine fish.

Genetic resources such as microsatellite markers were used for species identification of *Sebastes* species over the last two decades (Roques et al., 1999, 2001, 2002; Valentin et al., 2014). Here, we found that only two SNPs were needed to accurately identify the two species. We also found that at least a subset of 2,000 SNPs could discriminate redfish *S. mentella* deep and shallow ecotype. This subset of 2,000 SNPs outperformed microsatellite markers for delineating ecotypes and improved assignment test success (i.e., ecotypes of *S. mentella*) as shown in a wide range of species with management or conservation concerns such as harbor porpoise (*Phocoena phocoena;* Lah et al., 2016), the brown trout (*Salmo trutta*; Lemopoulos et al., 2019), the Atlantic salmon (*Salmo salar*; Moore et al., 2014), and the great scallop (*Pecten maximus*; Vendrami et al., 2017). Therefore, a next step could be to develop a genotype assay (e.g., using Kaspar, Fluidigm, AmpliSeq, or Sequenom technologies)—based on the selection of these highly divergent SNP markers combined with an individual assignment test approach—in order to provide an efficient tool for traceability as previously validated for several marine resources in Europe (Nielsen et al., 2012). The DNA assay developed for *Sebastes* spp. would serve to distinguish the two species and three ecotypes within the fish catches in order to assess the true impacts of the redfish fishery in Canada. Furthermore, a surprising high assignment success was achieved for *S. fasciatus* populations, with up to 96.5% of success while the vast majority of the sampling locations always exhibit a mixing proportion of different genetic clusters. This surprising result indicates that more samples might be required for enhancing the resolution of population structure and assignment test and eventually develop similar DNA chip for *S. fasciatus* populations.

## Supporting information

Fig S1

Fig S3

Fig S5

Supplementary Material

## Data Availability

Data for this study are available in DRYAD (https://datadryad.org) after the manuscript is accepted for publication. Scripts on population genomics and spatial ecology are available on the GitHub page of Laura M. Benestan and Quentin Rougemont.
