# Supplementary figures and images for: Population genomics and history of speciation reveal fishery management gaps in two related redfish species (Sebastes mentella and Sebastes fasciatus)
Source: Evol Appl. 2020 Dec 14;14(2):588–606. doi: 10.1111/eva.13143 (PMC7896722; doi:10.1111/eva.13143)

Number of individuals

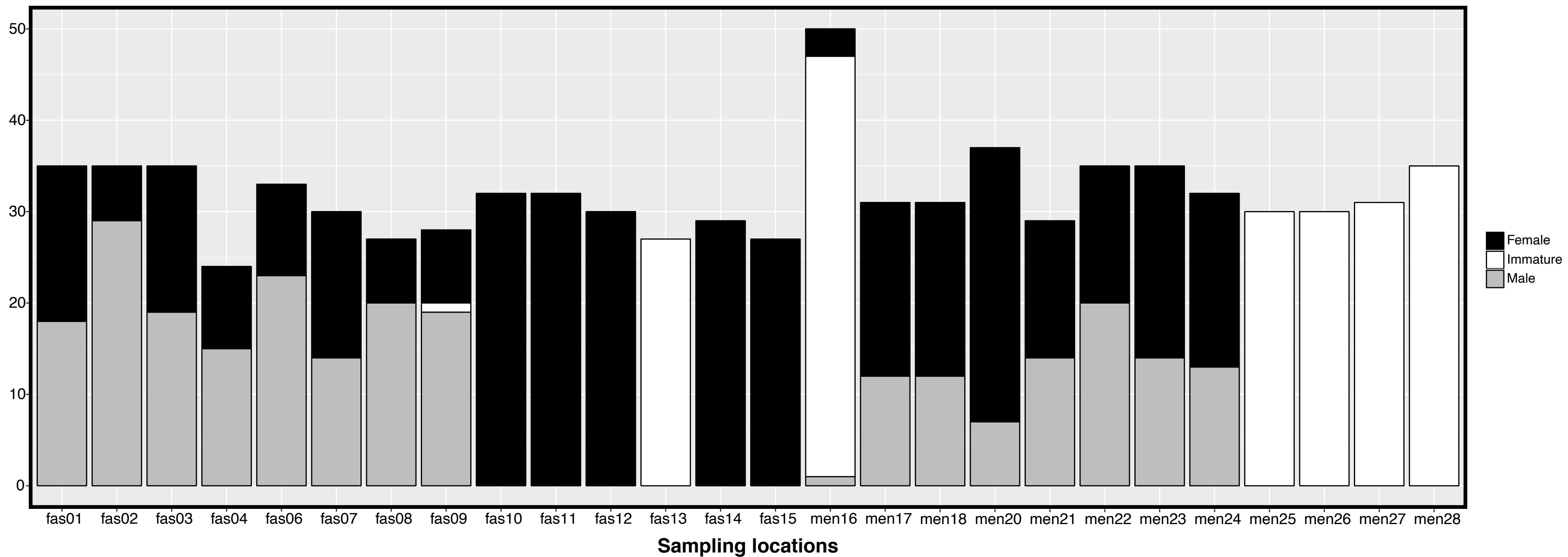

Supplement: Supplementary file 1 — Fig S1 [file EVA-14-588-s001.pdf]

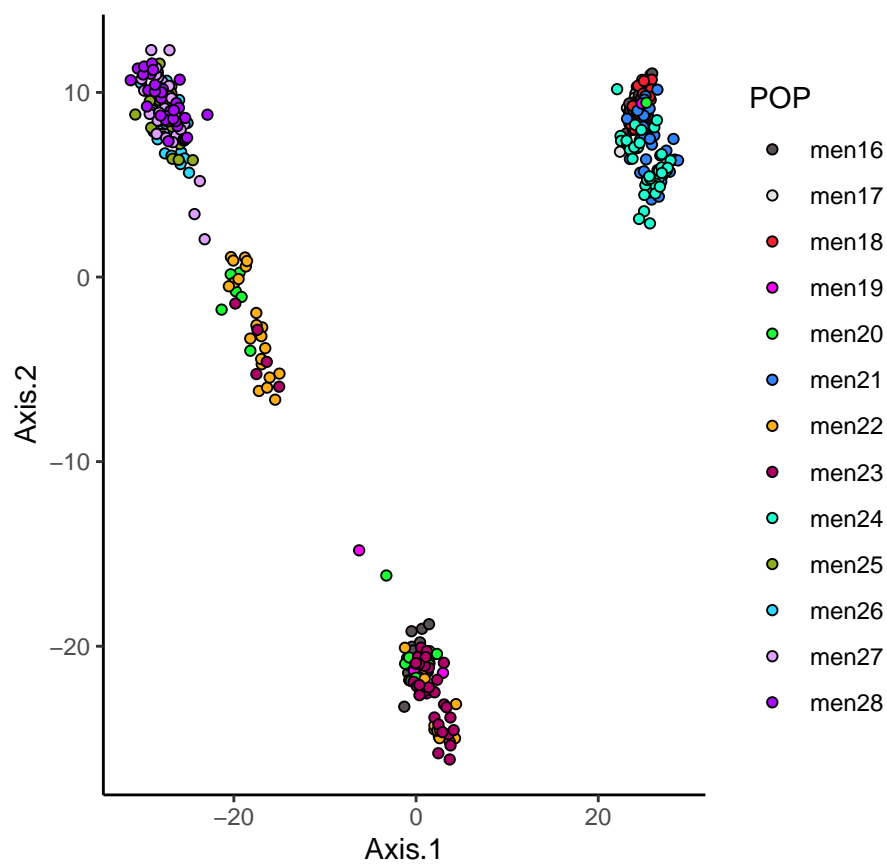

Supplement: Supplementary file 2 — Fig S3 [file EVA-14-588-s002.pdf]

Sampling location A

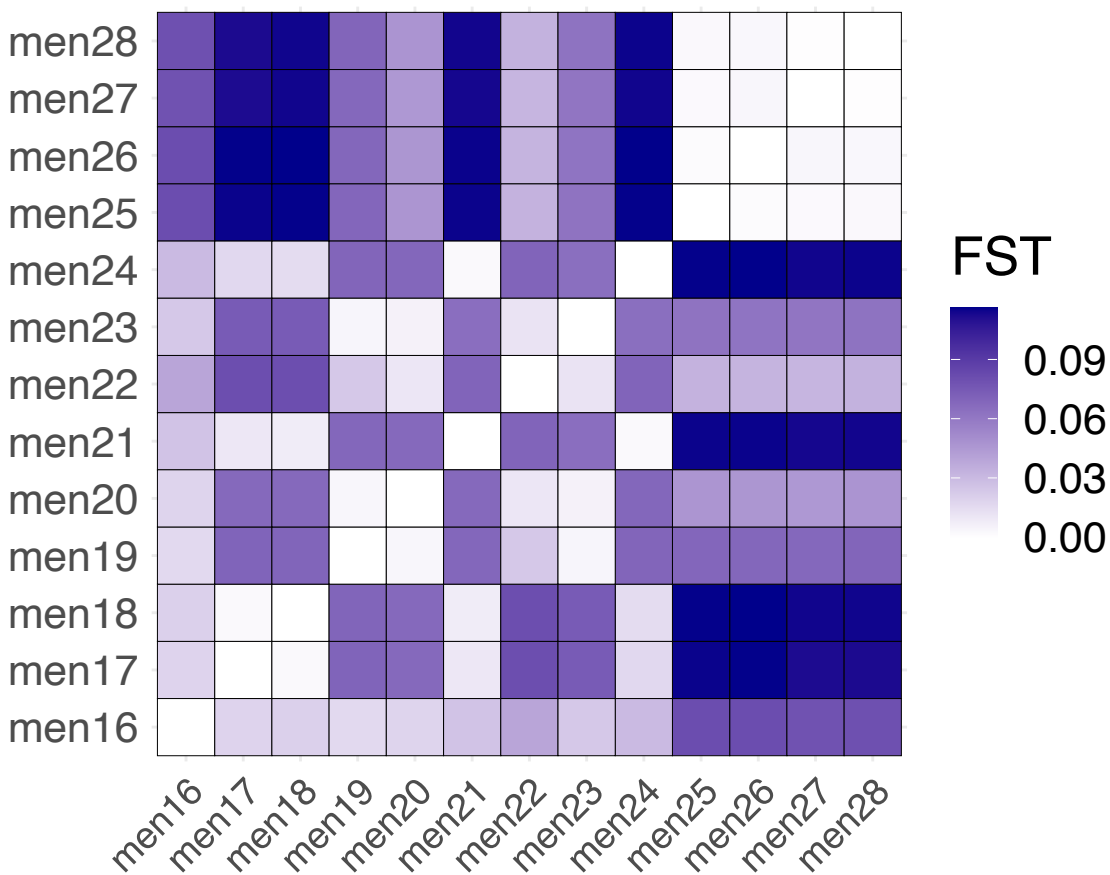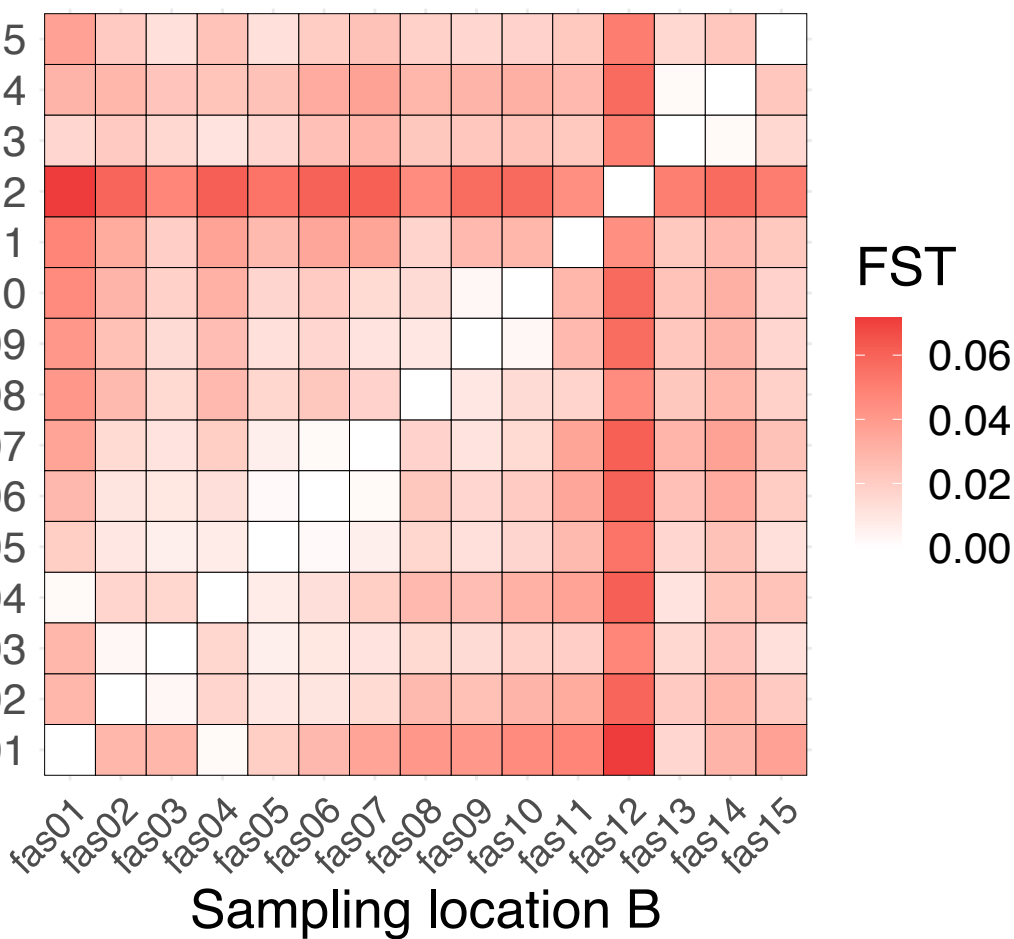

Supplement: Supplementary file 3 — Fig S5 [file EVA-14-588-s003.pdf]
