# Supplementary material for: Population genomics and history of speciation reveal fishery management gaps in two related redfish species (Sebastes mentella and Sebastes fasciatus)
Source: Evol Appl. 2020 Dec 14;14(2):588–606. doi: 10.1111/eva.13143 (PMC7896722; doi:10.1111/eva.13143)
Supplement: Supplementary file 4 — Supplementary Material [file EVA-14-588-s004.docx]

**Fig. S1.**

**Fig. S2.**

**
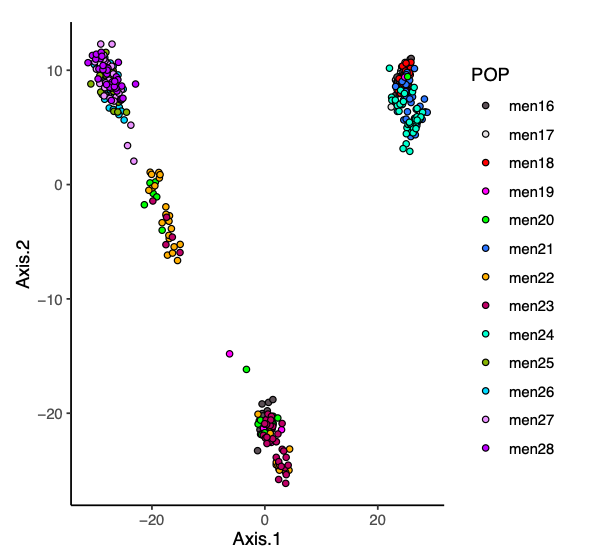
**

**Fig. S3.**

**Fig. S4.**

**Fig. S5.**

**
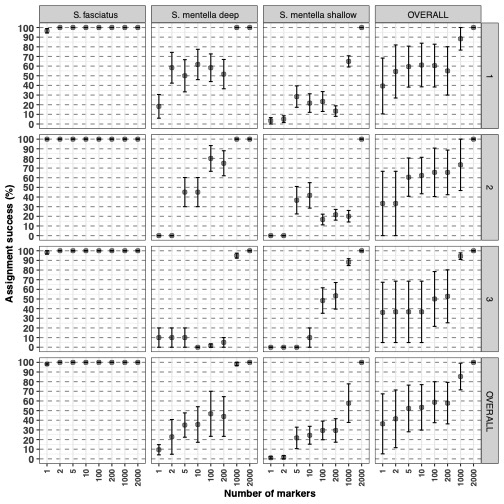
**

**Fig. S6.**

**Fig. S7**.

**Fig. S8**.

**Fig. S9.**

**Table S1.** Sampling location and geographic coordinates (latitude and longitude) of each individual used in the db-RDA analysis.

| **Label** | **Sampling location** | **Latitude** | **Longitude** |
| --- | --- | --- | --- |
| fas15_red081 | fas15 | 42.32 | -67.07 |
| fas15_red082 | fas15 | 42.32 | -67.07 |
| fas15_red083 | fas15 | 42.32 | -67.07 |
| fas15_red084 | fas15 | 42.32 | -67.07 |
| fas15_red085 | fas15 | 42.32 | -67.07 |
| fas15_red086 | fas15 | 42.32 | -67.07 |
| fas15_red087 | fas15 | 42.32 | -67.07 |
| fas15_red088 | fas15 | 42.32 | -67.07 |
| fas15_red089 | fas15 | 42.32 | -67.07 |
| fas15_red090 | fas15 | 42.32 | -67.07 |
| fas15_red091 | fas15 | 42.32 | -67.07 |
| fas15_red092 | fas15 | 42.32 | -67.07 |
| fas15_red093 | fas15 | 42.32 | -67.07 |
| fas15_red094 | fas15 | 42.32 | -67.07 |
| fas15_red095 | fas15 | 42.32 | -67.07 |
| fas15_red096 | fas15 | 42.32 | -67.07 |
| fas15_red097 | fas15 | 42.32 | -67.07 |
| fas15_red098 | fas15 | 42.32 | -67.07 |
| fas15_red099 | fas15 | 42.32 | -67.07 |
| fas15_red100 | fas15 | 42.32 | -67.07 |
| fas15_red101 | fas15 | 42.32 | -67.07 |
| fas15_red102 | fas15 | 42.32 | -67.07 |
| fas15_red103 | fas15 | 42.32 | -67.07 |
| fas15_red104 | fas15 | 42.32 | -67.07 |
| fas15_red105 | fas15 | 42.32 | -67.07 |
| fas15_red106 | fas15 | 42.32 | -67.07 |
| fas15_red107 | fas15 | 42.32 | -67.07 |
| fas12_red108 | fas12 | 49.57 | -57.95 |
| fas12_red109 | fas12 | 49.57 | -57.95 |
| fas12_red110 | fas12 | 49.57 | -57.95 |
| fas12_red111 | fas12 | 49.57 | -57.95 |
| fas12_red112 | fas12 | 49.57 | -57.95 |
| fas12_red113 | fas12 | 49.57 | -57.95 |
| fas12_red114 | fas12 | 49.57 | -57.95 |
| fas12_red115 | fas12 | 49.57 | -57.95 |
| fas12_red116 | fas12 | 49.57 | -57.95 |
| fas12_red117 | fas12 | 49.57 | -57.95 |
| fas12_red118 | fas12 | 49.57 | -57.95 |
| fas12_red119 | fas12 | 49.57 | -57.95 |
| fas12_red120 | fas12 | 49.57 | -57.95 |
| fas12_red121 | fas12 | 49.57 | -57.95 |
| fas12_red122 | fas12 | 49.57 | -57.95 |
| fas12_red123 | fas12 | 49.57 | -57.95 |
| fas12_red124 | fas12 | 49.57 | -57.95 |
| fas12_red125 | fas12 | 49.57 | -57.95 |
| fas12_red126 | fas12 | 49.57 | -57.95 |
| fas12_red127 | fas12 | 49.57 | -57.95 |
| fas12_red128 | fas12 | 49.57 | -57.95 |
| fas12_red129 | fas12 | 49.57 | -57.95 |
| fas12_red130 | fas12 | 49.57 | -57.95 |
| fas12_red131 | fas12 | 49.57 | -57.95 |
| fas12_red132 | fas12 | 49.57 | -57.95 |
| fas12_red133 | fas12 | 49.57 | -57.95 |
| fas12_red134 | fas12 | 49.57 | -57.95 |
| fas12_red135 | fas12 | 49.57 | -57.95 |
| fas12_red136 | fas12 | 49.57 | -57.95 |
| fas12_red137 | fas12 | 49.57 | -57.95 |
| fas13_red139 | fas13 | 49.82 | -65.41 |
| fas13_red144 | fas13 | 49.82 | -65.41 |
| fas13_red145 | fas13 | 49.82 | -65.41 |
| fas13_red146 | fas13 | 49.82 | -65.41 |
| fas13_red148 | fas13 | 49.82 | -65.41 |
| fas13_red150 | fas13 | 49.82 | -65.41 |
| fas13_red151 | fas13 | 49.82 | -65.41 |
| fas13_red152 | fas13 | 49.82 | -65.41 |
| fas13_red153 | fas13 | 49.82 | -65.41 |
| fas13_red154 | fas13 | 49.82 | -65.41 |
| fas13_red155 | fas13 | 49.82 | -65.41 |
| fas13_red162 | fas13 | 49.82 | -65.41 |
| fas13_red163 | fas13 | 49.82 | -65.41 |
| fas13_red165 | fas13 | 49.82 | -65.41 |
| fas13_red166 | fas13 | 49.82 | -65.41 |
| fas13_red167 | fas13 | 49.82 | -65.41 |
| fas13_red169 | fas13 | 49.82 | -65.41 |
| fas13_red170 | fas13 | 49.82 | -65.41 |
| fas13_red174 | fas13 | 49.82 | -65.41 |
| fas11_red193 | fas11 | 48.3 | -59.74 |
| fas11_red194 | fas11 | 48.3 | -59.74 |
| fas11_red195 | fas11 | 48.3 | -59.74 |
| fas11_red196 | fas11 | 48.3 | -59.74 |
| fas11_red197 | fas11 | 48.3 | -59.74 |
| fas11_red198 | fas11 | 48.3 | -59.74 |
| fas11_red199 | fas11 | 48.3 | -59.74 |
| fas11_red200 | fas11 | 48.3 | -59.74 |
| fas11_red201 | fas11 | 48.3 | -59.74 |
| fas11_red202 | fas11 | 48.3 | -59.74 |
| fas11_red203 | fas11 | 48.3 | -59.74 |
| fas11_red204 | fas11 | 48.3 | -59.74 |
| fas11_red205 | fas11 | 48.3 | -59.74 |
| fas11_red206 | fas11 | 48.3 | -59.74 |
| fas11_red207 | fas11 | 48.3 | -59.74 |
| fas11_red208 | fas11 | 48.3 | -59.74 |
| fas11_red209 | fas11 | 48.3 | -59.74 |
| fas11_red210 | fas11 | 48.3 | -59.74 |
| fas11_red211 | fas11 | 48.3 | -59.74 |
| fas11_red212 | fas11 | 48.3 | -59.74 |
| fas11_red213 | fas11 | 48.3 | -59.74 |
| fas11_red214 | fas11 | 48.3 | -59.74 |
| fas11_red215 | fas11 | 48.3 | -59.74 |
| fas11_red216 | fas11 | 48.3 | -59.74 |
| fas11_red217 | fas11 | 48.3 | -59.74 |
| fas11_red218 | fas11 | 48.3 | -59.74 |
| fas11_red219 | fas11 | 48.3 | -59.74 |
| fas11_red220 | fas11 | 48.3 | -59.74 |
| fas11_red221 | fas11 | 48.3 | -59.74 |
| fas11_red222 | fas11 | 48.3 | -59.74 |
| fas11_red223 | fas11 | 48.3 | -59.74 |
| fas11_red224 | fas11 | 48.3 | -59.74 |
| fas10_red225 | fas10 | 44.87 | -56.03 |
| fas10_red226 | fas10 | 44.87 | -56.03 |
| fas10_red227 | fas10 | 44.87 | -56.03 |
| fas10_red228 | fas10 | 44.87 | -56.03 |
| fas10_red229 | fas10 | 44.87 | -56.03 |
| fas10_red230 | fas10 | 44.87 | -56.03 |
| fas10_red231 | fas10 | 44.87 | -56.03 |
| fas10_red232 | fas10 | 44.87 | -56.03 |
| fas10_red233 | fas10 | 44.87 | -56.03 |
| fas10_red234 | fas10 | 44.87 | -56.03 |
| fas10_red235 | fas10 | 44.87 | -56.03 |
| fas10_red236 | fas10 | 44.87 | -56.03 |
| fas10_red237 | fas10 | 44.87 | -56.03 |
| fas10_red238 | fas10 | 44.87 | -56.03 |
| fas10_red239 | fas10 | 44.87 | -56.03 |
| fas10_red240 | fas10 | 44.87 | -56.03 |
| fas10_red241 | fas10 | 44.87 | -56.03 |
| fas10_red242 | fas10 | 44.87 | -56.03 |
| fas10_red243 | fas10 | 44.87 | -56.03 |
| fas10_red244 | fas10 | 44.87 | -56.03 |
| fas10_red245 | fas10 | 44.87 | -56.03 |
| fas10_red246 | fas10 | 44.87 | -56.03 |
| fas10_red247 | fas10 | 44.87 | -56.03 |
| fas10_red248 | fas10 | 44.87 | -56.03 |
| fas10_red249 | fas10 | 44.87 | -56.03 |
| fas10_red250 | fas10 | 44.87 | -56.03 |
| fas10_red251 | fas10 | 44.87 | -56.03 |
| fas10_red252 | fas10 | 44.87 | -56.03 |
| fas10_red253 | fas10 | 44.87 | -56.03 |
| fas10_red254 | fas10 | 44.87 | -56.03 |
| fas10_red255 | fas10 | 44.87 | -56.03 |
| fas10_red256 | fas10 | 44.87 | -56.03 |
| fas14_red257 | fas14 | 49.76 | -59.18 |
| fas14_red258 | fas14 | 49.76 | -59.18 |
| fas14_red259 | fas14 | 49.76 | -59.18 |
| fas14_red260 | fas14 | 49.76 | -59.18 |
| fas14_red261 | fas14 | 49.76 | -59.18 |
| fas14_red262 | fas14 | 49.76 | -59.18 |
| fas14_red263 | fas14 | 49.76 | -59.18 |
| fas14_red264 | fas14 | 49.76 | -59.18 |
| fas14_red265 | fas14 | 49.76 | -59.18 |
| fas14_red266 | fas14 | 49.76 | -59.18 |
| fas14_red267 | fas14 | 49.76 | -59.18 |
| fas14_red268 | fas14 | 49.76 | -59.18 |
| fas14_red269 | fas14 | 49.76 | -59.18 |
| fas14_red270 | fas14 | 49.76 | -59.18 |
| fas14_red271 | fas14 | 49.76 | -59.18 |
| fas14_red272 | fas14 | 49.76 | -59.18 |
| fas14_red273 | fas14 | 49.76 | -59.18 |
| fas14_red274 | fas14 | 49.76 | -59.18 |
| fas14_red275 | fas14 | 49.76 | -59.18 |
| fas14_red276 | fas14 | 49.76 | -59.18 |
| fas14_red277 | fas14 | 49.76 | -59.18 |
| fas14_red278 | fas14 | 49.76 | -59.18 |
| fas14_red279 | fas14 | 49.76 | -59.18 |
| fas14_red280 | fas14 | 49.76 | -59.18 |
| fas14_red281 | fas14 | 49.76 | -59.18 |
| fas14_red282 | fas14 | 49.76 | -59.18 |
| fas14_red283 | fas14 | 49.76 | -59.18 |
| fas14_red284 | fas14 | 49.76 | -59.18 |
| fas14_red285 | fas14 | 49.76 | -59.18 |
| fas14_red286 | fas14 | 49.76 | -59.18 |
| fas13_red287 | fas13 | 49.17 | -60.15 |
| fas13_red288 | fas13 | 50.25 | -58.7 |
| fas13_red290 | fas13 | 50.25 | -58.7 |
| fas13_red292 | fas13 | 50.13 | -58.08 |
| fas13_red297 | fas13 | 50.13 | -58.08 |
| fas13_red308 | fas13 | 50.13 | -58.08 |
| fas04_red325 | fas04 | 46.51 | -47.04 |
| fas04_red326 | fas04 | 46.51 | -47.04 |
| fas04_red327 | fas04 | 46.51 | -47.04 |
| fas04_red328 | fas04 | 47.01 | -47.23 |
| fas04_red329 | fas04 | 47.01 | -47.23 |
| fas04_red331 | fas04 | 47.01 | -47.23 |
| fas04_red334 | fas04 | 50.27 | -50.53 |
| fas04_red335 | fas04 | 50.27 | -50.53 |
| fas04_red336 | fas04 | 50.27 | -50.53 |
| fas04_red337 | fas04 | 50.27 | -50.53 |
| fas04_red338 | fas04 | 50.27 | -50.53 |
| fas04_red340 | fas04 | 50.64 | -50.67 |
| fas04_red341 | fas04 | 50.64 | -50.67 |
| fas04_red342 | fas04 | 50.64 | -50.67 |
| fas04_red343 | fas04 | 50.64 | -50.67 |
| fas04_red344 | fas04 | 50.64 | -50.67 |
| fas04_red345 | fas04 | 50.64 | -50.67 |
| fas04_red346 | fas04 | 50.64 | -50.67 |
| fas04_red347 | fas04 | 50.64 | -50.67 |
| fas04_red348 | fas04 | 50.64 | -50.67 |
| fas04_red349 | fas04 | 50.64 | -50.67 |
| fas04_red350 | fas04 | 50.64 | -50.67 |
| fas01_red351 | fas01 | 52.72 | -53.77 |
| fas01_red352 | fas01 | 52.72 | -53.77 |
| fas01_red353 | fas01 | 52.72 | -53.77 |
| fas01_red354 | fas01 | 52.72 | -53.77 |
| fas01_red355 | fas01 | 52.72 | -53.77 |
| fas01_red356 | fas01 | 52.72 | -53.77 |
| fas01_red357 | fas01 | 52.72 | -53.77 |
| fas01_red358 | fas01 | 52.72 | -53.77 |
| fas01_red359 | fas01 | 52.72 | -53.77 |
| fas01_red360 | fas01 | 52.72 | -53.77 |
| fas01_red361 | fas01 | 52.72 | -53.77 |
| fas01_red362 | fas01 | 52.72 | -53.77 |
| fas01_red363 | fas01 | 52.72 | -53.77 |
| fas01_red364 | fas01 | 52.72 | -53.77 |
| fas04_red365 | fas04 | 52.72 | -53.77 |
| fas01_red366 | fas01 | 52.72 | -53.77 |
| fas01_red367 | fas01 | 52.72 | -53.77 |
| fas01_red368 | fas01 | 52.72 | -53.77 |
| fas04_red369 | fas04 | 52.72 | -53.77 |
| fas01_red370 | fas01 | 52.72 | -53.77 |
| fas04_red371 | fas04 | 52.72 | -53.77 |
| fas01_red372 | fas01 | 52.72 | -53.77 |
| fas04_red373 | fas04 | 52.72 | -53.77 |
| fas01_red374 | fas01 | 53.14 | -54.38 |
| fas01_red375 | fas01 | 53.14 | -54.38 |
| fas01_red376 | fas01 | 53.14 | -54.38 |
| fas04_red377 | fas04 | 53.14 | -54.38 |
| fas01_red378 | fas01 | 53.14 | -54.38 |
| fas01_red379 | fas01 | 53.14 | -54.38 |
| fas01_red380 | fas01 | 53.14 | -54.38 |
| fas01_red381 | fas01 | 53.14 | -54.38 |
| fas01_red382 | fas01 | 53.14 | -54.38 |
| fas04_red383 | fas04 | 53.14 | -54.38 |
| fas01_red384 | fas01 | 53.14 | -54.38 |
| fas01_red385 | fas01 | 53.14 | -54.38 |
| fas05_red386 | fas05 | 48.04 | -48.51 |
| fas05_red387 | fas05 | 48.04 | -48.51 |
| fas05_red388 | fas05 | 48.04 | -48.51 |
| fas05_red389 | fas05 | 48.04 | -48.51 |
| fas05_red390 | fas05 | 48.04 | -48.51 |
| fas05_red391 | fas05 | 48.04 | -48.51 |
| fas05_red392 | fas05 | 48.04 | -48.51 |
| fas05_red393 | fas05 | 48.04 | -48.51 |
| fas05_red394 | fas05 | 48.04 | -48.51 |
| fas05_red395 | fas05 | 48.04 | -48.51 |
| fas05_red396 | fas05 | 48.04 | -48.51 |
| fas05_red397 | fas05 | 48.04 | -48.51 |
| fas05_red398 | fas05 | 48.04 | -48.51 |
| fas05_red399 | fas05 | 48.04 | -48.51 |
| fas05_red400 | fas05 | 48.04 | -48.51 |
| fas05_red401 | fas05 | 48.31 | -49.08 |
| fas05_red402 | fas05 | 48.31 | -49.08 |
| fas05_red403 | fas05 | 48.31 | -49.08 |
| fas05_red404 | fas05 | 48.31 | -49.08 |
| fas05_red405 | fas05 | 48.31 | -49.08 |
| fas05_red406 | fas05 | 48.31 | -49.08 |
| fas05_red407 | fas05 | 48.31 | -49.08 |
| fas05_red408 | fas05 | 48.31 | -49.08 |
| fas05_red409 | fas05 | 48.31 | -49.08 |
| fas05_red410 | fas05 | 48.31 | -49.08 |
| fas05_red411 | fas05 | 48.36 | -49.14 |
| fas05_red412 | fas05 | 48.36 | -49.14 |
| fas05_red413 | fas05 | 48.36 | -49.14 |
| fas05_red414 | fas05 | 48.36 | -49.14 |
| fas05_red415 | fas05 | 48.31 | -49.34 |
| fas05_red416 | fas05 | 48.31 | -49.34 |
| fas05_red417 | fas05 | 48.31 | -49.34 |
| fas05_red418 | fas05 | 48.31 | -49.34 |
| fas05_red419 | fas05 | 48.31 | -49.34 |
| fas05_red420 | fas05 | 48.31 | -49.34 |
| fas06_red421 | fas06 | 46.05 | -47.68 |
| fas06_red422 | fas06 | 46.05 | -47.68 |
| fas06_red423 | fas06 | 46.05 | -47.68 |
| fas06_red424 | fas06 | 46.05 | -47.68 |
| fas06_red425 | fas06 | 46.05 | -47.68 |
| fas06_red426 | fas06 | 46.05 | -47.68 |
| fas06_red427 | fas06 | 46.05 | -47.68 |
| fas06_red428 | fas06 | 46.05 | -47.68 |
| fas06_red429 | fas06 | 46.05 | -47.68 |
| fas06_red430 | fas06 | 46.05 | -47.68 |
| fas06_red431 | fas06 | 46.05 | -47.68 |
| fas06_red432 | fas06 | 46.05 | -47.68 |
| fas06_red433 | fas06 | 46.36 | -47.27 |
| fas06_red434 | fas06 | 46.36 | -47.27 |
| fas06_red435 | fas06 | 46.36 | -47.27 |
| fas06_red436 | fas06 | 46.36 | -47.27 |
| fas06_red437 | fas06 | 46.36 | -47.27 |
| fas06_red438 | fas06 | 46.36 | -47.27 |
| fas06_red439 | fas06 | 46.36 | -47.27 |
| fas06_red440 | fas06 | 46.36 | -47.27 |
| fas06_red441 | fas06 | 46.36 | -47.27 |
| fas06_red442 | fas06 | 46.36 | -47.27 |
| fas06_red443 | fas06 | 46.36 | -47.27 |
| fas06_red444 | fas06 | 46.36 | -47.27 |
| fas06_red445 | fas06 | 46.36 | -47.27 |
| fas06_red446 | fas06 | 46.36 | -47.27 |
| fas06_red447 | fas06 | 46.36 | -47.27 |
| fas06_red448 | fas06 | 46.36 | -47.27 |
| fas06_red449 | fas06 | 46.36 | -47.27 |
| fas06_red450 | fas06 | 46.36 | -47.27 |
| fas06_red451 | fas06 | 46.36 | -47.27 |
| fas06_red452 | fas06 | 46.36 | -47.27 |
| fas06_red453 | fas06 | 46.36 | -47.27 |
| fas06_red454 | fas06 | 46.36 | -47.27 |
| fas06_red455 | fas06 | 46.36 | -47.27 |
| fas03_red596 | fas03 | 48.17 | -53.64 |
| fas03_red597 | fas03 | 48.17 | -53.64 |
| fas03_red598 | fas03 | 48.17 | -53.64 |
| fas03_red599 | fas03 | 48.17 | -53.64 |
| fas03_red600 | fas03 | 48.17 | -53.64 |
| fas03_red601 | fas03 | 48.17 | -53.64 |
| fas03_red602 | fas03 | 48.17 | -53.64 |
| fas03_red603 | fas03 | 48.17 | -53.64 |
| fas03_red604 | fas03 | 48.17 | -53.64 |
| fas03_red605 | fas03 | 48.17 | -53.64 |
| fas03_red606 | fas03 | 48.17 | -53.64 |
| fas03_red607 | fas03 | 48.17 | -53.64 |
| fas03_red608 | fas03 | 48.17 | -53.64 |
| fas03_red609 | fas03 | 48.17 | -53.64 |
| fas03_red610 | fas03 | 48.17 | -53.64 |
| fas03_red611 | fas03 | 48.17 | -53.64 |
| fas03_red612 | fas03 | 48.17 | -53.64 |
| fas03_red613 | fas03 | 48.17 | -53.64 |
| fas03_red614 | fas03 | 48.17 | -53.64 |
| fas03_red615 | fas03 | 48.17 | -53.64 |
| fas03_red616 | fas03 | 48.17 | -53.64 |
| fas03_red617 | fas03 | 48.17 | -53.64 |
| fas03_red618 | fas03 | 48.17 | -53.64 |
| fas03_red619 | fas03 | 48.17 | -53.64 |
| fas03_red620 | fas03 | 48.17 | -53.64 |
| fas03_red621 | fas03 | 48.17 | -53.64 |
| fas03_red622 | fas03 | 48.17 | -53.64 |
| fas03_red623 | fas03 | 48.17 | -53.64 |
| fas03_red624 | fas03 | 48.17 | -53.64 |
| fas03_red625 | fas03 | 48.17 | -53.64 |
| fas03_red626 | fas03 | 48.17 | -53.64 |
| fas03_red627 | fas03 | 48.17 | -53.64 |
| fas03_red628 | fas03 | 48.17 | -53.64 |
| fas03_red629 | fas03 | 48.17 | -53.64 |
| fas03_red630 | fas03 | 48.17 | -53.64 |
| fas02_red631 | fas02 | 49.5 | -56.17 |
| fas02_red632 | fas02 | 49.5 | -56.17 |
| fas02_red633 | fas02 | 49.5 | -56.17 |
| fas02_red634 | fas02 | 49.5 | -56.17 |
| fas02_red635 | fas02 | 49.5 | -56.17 |
| fas02_red636 | fas02 | 49.5 | -56.17 |
| fas02_red637 | fas02 | 49.5 | -56.17 |
| fas02_red638 | fas02 | 49.5 | -56.17 |
| fas02_red639 | fas02 | 49.5 | -56.17 |
| fas02_red640 | fas02 | 49.5 | -56.17 |
| fas02_red641 | fas02 | 49.5 | -56.17 |
| fas02_red642 | fas02 | 49.5 | -56.17 |
| fas02_red643 | fas02 | 49.5 | -56.17 |
| fas02_red644 | fas02 | 49.5 | -56.17 |
| fas02_red645 | fas02 | 49.5 | -56.17 |
| fas02_red646 | fas02 | 49.5 | -56.17 |
| fas02_red647 | fas02 | 49.5 | -56.17 |
| fas02_red648 | fas02 | 49.5 | -56.17 |
| fas02_red649 | fas02 | 49.5 | -56.17 |
| fas02_red650 | fas02 | 49.5 | -56.17 |
| fas02_red651 | fas02 | 49.5 | -56.17 |
| fas02_red652 | fas02 | 49.5 | -56.17 |
| fas02_red653 | fas02 | 49.5 | -56.17 |
| fas02_red654 | fas02 | 49.5 | -56.17 |
| fas02_red655 | fas02 | 49.5 | -56.17 |
| fas02_red656 | fas02 | 49.5 | -56.17 |
| fas02_red657 | fas02 | 49.5 | -56.17 |
| fas02_red658 | fas02 | 49.5 | -56.17 |
| fas02_red659 | fas02 | 49.5 | -56.17 |
| fas02_red660 | fas02 | 49.5 | -56.17 |
| fas02_red661 | fas02 | 49.5 | -56.17 |
| fas02_red662 | fas02 | 49.5 | -56.17 |
| fas02_red663 | fas02 | 49.5 | -56.17 |
| fas02_red664 | fas02 | 49.5 | -56.17 |
| fas07_red665 | fas07 | 49.5 | -56.17 |
| fas07_red666 | fas07 | 44.47 | -49.02 |
| fas07_red667 | fas07 | 44.47 | -49.02 |
| fas07_red668 | fas07 | 44.47 | -49.02 |
| fas07_red669 | fas07 | 44.47 | -49.02 |
| fas07_red670 | fas07 | 44.47 | -49.02 |
| fas07_red671 | fas07 | 44.47 | -49.02 |
| fas07_red672 | fas07 | 44.47 | -49.02 |
| fas07_red673 | fas07 | 44.47 | -49.02 |
| fas07_red674 | fas07 | 44.47 | -49.02 |
| fas07_red675 | fas07 | 44.47 | -49.02 |
| fas07_red676 | fas07 | 44.47 | -49.02 |
| fas07_red677 | fas07 | 44.47 | -49.02 |
| fas07_red678 | fas07 | 44.47 | -49.02 |
| fas07_red679 | fas07 | 44.47 | -49.02 |
| fas07_red680 | fas07 | 44.47 | -49.02 |
| fas07_red682 | fas07 | 43.06 | -50.98 |
| fas07_red683 | fas07 | 43.06 | -50.98 |
| fas07_red684 | fas07 | 43.06 | -50.98 |
| fas07_red685 | fas07 | 43.06 | -50.98 |
| fas07_red686 | fas07 | 43.06 | -50.98 |
| fas07_red687 | fas07 | 43.06 | -50.98 |
| fas07_red688 | fas07 | 43.06 | -50.98 |
| fas07_red689 | fas07 | 43.06 | -50.98 |
| fas07_red690 | fas07 | 43.06 | -50.98 |
| fas07_red691 | fas07 | 43.06 | -50.98 |
| fas07_red692 | fas07 | 43.06 | -50.98 |
| fas07_red693 | fas07 | 43.06 | -50.98 |
| fas07_red694 | fas07 | 43.06 | -50.98 |
| fas07_red695 | fas07 | 43.06 | -50.98 |
| fas09_red700 | fas09 | 44.93 | -54.44 |
| fas09_red702 | fas09 | 44.93 | -54.44 |
| fas09_red703 | fas09 | 44.93 | -54.44 |
| fas09_red704 | fas09 | 44.93 | -54.44 |
| fas09_red706 | fas09 | 44.93 | -54.44 |
| fas09_red707 | fas09 | 44.93 | -54.44 |
| fas09_red708 | fas09 | 44.93 | -54.44 |
| fas09_red709 | fas09 | 44.93 | -54.44 |
| fas09_red710 | fas09 | 44.93 | -54.44 |
| fas09_red712 | fas09 | 44.53 | -53.96 |
| fas08_red714 | fas08 | 42.76 | -50.05 |
| fas08_red715 | fas08 | 42.76 | -50.05 |
| fas08_red716 | fas08 | 42.76 | -50.05 |
| fas08_red717 | fas08 | 42.76 | -50.05 |
| fas08_red718 | fas08 | 42.76 | -50.05 |
| fas08_red722 | fas08 | 42.76 | -50.05 |
| fas09_red790 | fas09 | 44.93 | -54.44 |
| fas09_red791 | fas09 | 44.93 | -54.44 |
| fas09_red792 | fas09 | 44.93 | -54.44 |
| fas09_red794 | fas09 | 44.93 | -54.44 |
| fas09_red795 | fas09 | 44.93 | -54.44 |
| fas09_red796 | fas09 | 44.93 | -54.44 |
| fas09_red797 | fas09 | 44.93 | -54.44 |
| fas09_red798 | fas09 | 44.53 | -53.96 |
| fas09_red800 | fas09 | 44.53 | -53.96 |
| fas09_red801 | fas09 | 44.53 | -53.96 |
| fas09_red802 | fas09 | 44.53 | -53.96 |
| fas09_red803 | fas09 | 44.53 | -53.96 |
| fas09_red804 | fas09 | 44.53 | -53.96 |
| fas08_red805 | fas08 | 42.76 | -50.05 |
| fas08_red807 | fas08 | 42.76 | -50.05 |
| fas08_red808 | fas08 | 42.76 | -50.05 |
| fas08_red809 | fas08 | 42.76 | -50.05 |
| fas08_red810 | fas08 | 42.76 | -50.05 |
| fas08_red812 | fas08 | 42.76 | -50.05 |
| fas08_red814 | fas08 | 42.76 | -50.05 |
| fas08_red815 | fas08 | 42.76 | -50.05 |
| fas08_red816 | fas08 | 42.76 | -50.05 |
| fas08_red817 | fas08 | 42.76 | -50.05 |
| fas08_red819 | fas08 | 42.76 | -50.05 |
| fas08_red820 | fas08 | 42.76 | -50.05 |
| fas08_red821 | fas08 | 42.76 | -50.05 |
| men17_red001 | men17 | 66.58 | -57.78 |
| men17_red002 | men17 | 66.58 | -57.78 |
| men17_red003 | men17 | 66.58 | -57.78 |
| men17_red004 | men17 | 66.58 | -57.78 |
| men17_red005 | men17 | 66.58 | -57.78 |
| men17_red006 | men17 | 66.58 | -57.78 |
| men17_red007 | men17 | 66.58 | -57.78 |
| men17_red008 | men17 | 66.58 | -57.78 |
| men17_red009 | men17 | 66.58 | -57.78 |
| men17_red010 | men17 | 66.58 | -57.78 |
| men17_red011 | men17 | 66.58 | -57.78 |
| men17_red012 | men17 | 66.58 | -57.78 |
| men17_red013 | men17 | 66.58 | -57.78 |
| men17_red014 | men17 | 66.58 | -57.78 |
| men17_red015 | men17 | 66.58 | -57.78 |
| men17_red016 | men17 | 66.58 | -57.78 |
| men17_red017 | men17 | 66.58 | -57.78 |
| men17_red018 | men17 | 66.58 | -57.78 |
| men17_red019 | men17 | 66.58 | -57.78 |
| men17_red020 | men17 | 66.58 | -57.78 |
| men17_red021 | men17 | 66.58 | -57.78 |
| men17_red022 | men17 | 66.58 | -57.78 |
| men17_red023 | men17 | 66.58 | -57.78 |
| men17_red024 | men17 | 66.58 | -57.78 |
| men17_red025 | men17 | 66.58 | -57.78 |
| men17_red026 | men17 | 66.58 | -57.78 |
| men17_red027 | men17 | 66.58 | -57.78 |
| men17_red028 | men17 | 66.58 | -57.78 |
| men17_red030 | men17 | 66.58 | -57.78 |
| men17_red031 | men17 | 66.58 | -57.78 |
| men16_red032 | men16 | 67.14 | -61.16 |
| men16_red033 | men16 | 67.14 | -61.16 |
| men16_red034 | men16 | 67.14 | -61.16 |
| men16_red035 | men16 | 67.14 | -61.16 |
| men16_red036 | men16 | 67.14 | -61.16 |
| men16_red037 | men16 | 67.14 | -61.16 |
| men16_red038 | men16 | 67.14 | -61.16 |
| men16_red039 | men16 | 67.14 | -61.16 |
| men16_red040 | men16 | 67.14 | -61.16 |
| men16_red041 | men16 | 67.14 | -61.16 |
| men16_red042 | men16 | 67.14 | -61.16 |
| men16_red043 | men16 | 67.14 | -61.16 |
| men16_red044 | men16 | 67.14 | -61.16 |
| men16_red045 | men16 | 67.14 | -61.16 |
| men16_red046 | men16 | 67.14 | -61.16 |
| men16_red047 | men16 | 67.14 | -61.16 |
| men16_red048 | men16 | 67.14 | -61.16 |
| men16_red049 | men16 | 67.14 | -61.16 |
| men16_red050 | men16 | 67.14 | -61.16 |
| men16_red051 | men16 | 67.14 | -61.16 |
| men16_red052 | men16 | 67.14 | -61.16 |
| men16_red053 | men16 | 67.14 | -61.16 |
| men16_red054 | men16 | 67.14 | -61.16 |
| men16_red055 | men16 | 67.14 | -61.16 |
| men16_red056 | men16 | 67.14 | -61.16 |
| men16_red057 | men16 | 67.14 | -61.16 |
| men16_red058 | men16 | 67.14 | -61.16 |
| men16_red059 | men16 | 67.14 | -61.16 |
| men16_red060 | men16 | 67.14 | -61.16 |
| men16_red061 | men16 | 67.14 | -61.16 |
| men16_red062 | men16 | 67.14 | -61.16 |
| men16_red063 | men16 | 67.14 | -61.16 |
| men16_red064 | men16 | 67.14 | -61.16 |
| men16_red065 | men16 | 67.14 | -61.16 |
| men16_red066 | men16 | 67.14 | -61.16 |
| men16_red067 | men16 | 67.14 | -61.16 |
| men16_red068 | men16 | 67.14 | -61.16 |
| men16_red069 | men16 | 67.14 | -61.16 |
| men16_red070 | men16 | 67.14 | -61.16 |
| men16_red071 | men16 | 67.14 | -61.16 |
| men16_red072 | men16 | 67.14 | -61.16 |
| men16_red073 | men16 | 67.14 | -61.16 |
| men16_red074 | men16 | 67.14 | -61.16 |
| men16_red075 | men16 | 67.14 | -61.16 |
| men16_red076 | men16 | 67.14 | -61.16 |
| men16_red078 | men16 | 67.14 | -61.16 |
| men16_red079 | men16 | 67.14 | -61.16 |
| men16_red080 | men16 | 67.14 | -61.16 |
| men28_red138 | men28 | 49.82 | -65.41 |
| men28_red140 | men28 | 49.82 | -65.41 |
| men28_red142 | men28 | 49.82 | -65.41 |
| men28_red143 | men28 | 49.82 | -65.41 |
| men28_red147 | men28 | 49.82 | -65.41 |
| men28_red149 | men28 | 49.82 | -65.41 |
| men28_red156 | men28 | 49.82 | -65.41 |
| men28_red157 | men28 | 49.82 | -65.41 |
| men28_red158 | men28 | 49.82 | -65.41 |
| men28_red159 | men28 | 49.82 | -65.41 |
| men28_red160 | men28 | 49.82 | -65.41 |
| men28_red161 | men28 | 49.82 | -65.41 |
| men28_red164 | men28 | 49.82 | -65.41 |
| men28_red168 | men28 | 49.82 | -65.41 |
| men28_red171 | men28 | 49.82 | -65.41 |
| men28_red172 | men28 | 49.82 | -65.41 |
| men28_red173 | men28 | 49.82 | -65.41 |
| men28_red175 | men28 | 49.82 | -65.41 |
| men28_red176 | men28 | 49.82 | -65.41 |
| men28_red177 | men28 | 49.89 | -66.68 |
| men28_red178 | men28 | 49.89 | -66.68 |
| men28_red179 | men28 | 49.89 | -66.68 |
| men28_red180 | men28 | 49.89 | -66.68 |
| men28_red182 | men28 | 49.89 | -66.68 |
| men28_red183 | men28 | 49.89 | -66.68 |
| men28_red184 | men28 | 49.89 | -66.68 |
| men28_red185 | men28 | 49.89 | -66.68 |
| men28_red186 | men28 | 49.89 | -66.68 |
| men28_red187 | men28 | 49.89 | -66.68 |
| men27_red188 | men27 | 50.13 | -58.08 |
| men27_red189 | men27 | 50.13 | -58.08 |
| men27_red190 | men27 | 50.13 | -58.08 |
| men27_red191 | men27 | 50.13 | -58.08 |
| men27_red192 | men27 | 50.13 | -58.08 |
| men27_red291 | men27 | 50.13 | -58.08 |
| men27_red293 | men27 | 50.13 | -58.08 |
| men27_red294 | men27 | 50.13 | -58.08 |
| men27_red295 | men27 | 50.13 | -58.08 |
| men27_red296 | men27 | 50.13 | -58.08 |
| men27_red298 | men27 | 50.13 | -58.08 |
| men27_red299 | men27 | 50.13 | -58.08 |
| men27_red300 | men27 | 50.13 | -58.08 |
| men27_red301 | men27 | 50.13 | -58.08 |
| men27_red302 | men27 | 50.13 | -58.08 |
| men27_red303 | men27 | 50.13 | -58.08 |
| men27_red304 | men27 | 50.13 | -58.08 |
| men27_red305 | men27 | 50.13 | -58.08 |
| men27_red306 | men27 | 50.13 | -58.08 |
| men27_red307 | men27 | 50.13 | -58.08 |
| men27_red309 | men27 | 50.13 | -58.08 |
| men27_red310 | men27 | 50.13 | -58.08 |
| men27_red311 | men27 | 50.13 | -58.08 |
| men27_red312 | men27 | 50.13 | -58.08 |
| men27_red313 | men27 | 50.13 | -58.08 |
| men27_red314 | men27 | 50.13 | -58.08 |
| men27_red315 | men27 | 50.13 | -58.08 |
| men27_red316 | men27 | 50.13 | -58.08 |
| men27_red317 | men27 | 50.13 | -58.08 |
| men27_red318 | men27 | 50.13 | -58.08 |
| men27_red319 | men27 | 50.13 | -58.08 |
| men28_red320 | men28 | 48.24 | -70.19 |
| men28_red321 | men28 | 48.32 | -70.85 |
| men28_red322 | men28 | 48.34 | -70.8 |
| men28_red323 | men28 | 48.35 | -70.87 |
| men22_red456 | men22 | 52.93 | -51.96 |
| men22_red457 | men22 | 52.93 | -51.96 |
| men22_red458 | men22 | 52.93 | -51.96 |
| men22_red459 | men22 | 52.93 | -51.96 |
| men22_red460 | men22 | 52.93 | -51.96 |
| men22_red461 | men22 | 52.93 | -51.96 |
| men22_red462 | men22 | 52.93 | -51.96 |
| men22_red463 | men22 | 52.93 | -51.96 |
| men22_red464 | men22 | 52.93 | -51.96 |
| men22_red465 | men22 | 52.93 | -51.96 |
| men22_red466 | men22 | 52.93 | -51.96 |
| men22_red467 | men22 | 52.93 | -51.96 |
| men22_red468 | men22 | 52.93 | -51.96 |
| men22_red469 | men22 | 52.93 | -51.96 |
| men22_red470 | men22 | 52.93 | -51.96 |
| men22_red471 | men22 | 52.93 | -51.96 |
| men22_red472 | men22 | 52.93 | -51.96 |
| men22_red473 | men22 | 52.93 | -51.96 |
| men22_red474 | men22 | 52.93 | -51.96 |
| men22_red475 | men22 | 52.93 | -51.96 |
| men22_red476 | men22 | 52.93 | -51.96 |
| men22_red477 | men22 | 52.93 | -51.96 |
| men22_red478 | men22 | 52.93 | -51.96 |
| men22_red479 | men22 | 52.93 | -51.96 |
| men22_red480 | men22 | 52.93 | -51.96 |
| men22_red481 | men22 | 52.93 | -51.96 |
| men22_red482 | men22 | 52.93 | -51.96 |
| men22_red483 | men22 | 52.93 | -51.96 |
| men22_red484 | men22 | 52.93 | -51.96 |
| men22_red485 | men22 | 52.93 | -51.96 |
| men22_red486 | men22 | 52.93 | -51.96 |
| men22_red487 | men22 | 52.93 | -51.96 |
| men22_red488 | men22 | 52.93 | -51.96 |
| men22_red489 | men22 | 52.93 | -51.96 |
| men22_red490 | men22 | 52.93 | -51.96 |
| men23_red491 | men23 | 48.04 | -48.51 |
| men23_red492 | men23 | 48.04 | -48.51 |
| men23_red493 | men23 | 48.41 | -49.46 |
| men23_red495 | men23 | 49.19 | -50.05 |
| men23_red496 | men23 | 49.19 | -50.05 |
| men23_red497 | men23 | 49.19 | -50.05 |
| men23_red498 | men23 | 49.19 | -50.05 |
| men23_red499 | men23 | 49.19 | -50.05 |
| men23_red500 | men23 | 50.64 | -50.67 |
| men23_red501 | men23 | 51.3 | -50.24 |
| men23_red502 | men23 | 51.3 | -50.24 |
| men23_red503 | men23 | 51.3 | -50.24 |
| men23_red504 | men23 | 51.3 | -50.24 |
| men23_red505 | men23 | 51.3 | -50.24 |
| men23_red506 | men23 | 51.3 | -50.24 |
| men23_red507 | men23 | 51.3 | -50.24 |
| men23_red508 | men23 | 51.3 | -50.24 |
| men23_red509 | men23 | 51.3 | -50.24 |
| men23_red510 | men23 | 51.3 | -50.24 |
| men23_red511 | men23 | 51.3 | -50.24 |
| men23_red512 | men23 | 51.3 | -50.24 |
| men23_red513 | men23 | 51.3 | -50.24 |
| men23_red514 | men23 | 51.3 | -50.24 |
| men23_red515 | men23 | 51.3 | -50.24 |
| men23_red516 | men23 | 51.3 | -50.24 |
| men23_red517 | men23 | 51.3 | -50.24 |
| men23_red518 | men23 | 51.3 | -50.24 |
| men23_red519 | men23 | 51.3 | -50.24 |
| men23_red520 | men23 | 51.3 | -50.24 |
| men23_red521 | men23 | 51.3 | -50.24 |
| men23_red522 | men23 | 51.3 | -50.24 |
| men23_red523 | men23 | 51.3 | -50.24 |
| men23_red524 | men23 | 51.3 | -50.24 |
| men23_red525 | men23 | 51.3 | -50.24 |
| men24_red526 | men24 | 46.36 | -47.27 |
| men24_red527 | men24 | 46.36 | -47.27 |
| men24_red528 | men24 | 46.36 | -47.27 |
| men24_red529 | men24 | 46.51 | -47.04 |
| men24_red530 | men24 | 46.51 | -47.04 |
| men24_red531 | men24 | 46.51 | -47.04 |
| men24_red532 | men24 | 46.51 | -47.04 |
| men24_red533 | men24 | 46.51 | -47.04 |
| men24_red534 | men24 | 46.51 | -47.04 |
| men24_red535 | men24 | 46.51 | -47.04 |
| men24_red536 | men24 | 47.01 | -47.23 |
| men24_red537 | men24 | 47.01 | -47.23 |
| men24_red538 | men24 | 47.01 | -47.23 |
| men24_red539 | men24 | 47.01 | -47.23 |
| men24_red540 | men24 | 47.01 | -47.23 |
| men24_red541 | men24 | 47.97 | -46.88 |
| men24_red542 | men24 | 47.97 | -46.88 |
| men24_red543 | men24 | 47.97 | -46.88 |
| men24_red544 | men24 | 47.97 | -46.88 |
| men24_red545 | men24 | 47.97 | -46.88 |
| men24_red546 | men24 | 47.97 | -46.88 |
| men24_red547 | men24 | 47.97 | -46.88 |
| men24_red548 | men24 | 47.97 | -46.88 |
| men24_red549 | men24 | 47.97 | -46.88 |
| men24_red550 | men24 | 47.97 | -46.88 |
| men24_red551 | men24 | 48.03 | -47.03 |
| men24_red552 | men24 | 48.03 | -47.03 |
| men24_red553 | men24 | 48.03 | -47.03 |
| men24_red554 | men24 | 48.03 | -47.03 |
| men24_red555 | men24 | 48.03 | -47.03 |
| men24_red556 | men24 | 48.03 | -47.03 |
| men24_red557 | men24 | 48.03 | -47.03 |
| men24_red558 | men24 | 48.03 | -47.03 |
| men24_red559 | men24 | 48.03 | -47.03 |
| men24_red560 | men24 | 48.03 | -47.03 |
| men24_red561 | men24 | 48.03 | -47.03 |
| men21_red562 | men21 | 52.93 | -51.96 |
| men21_red563 | men21 | 52.93 | -51.96 |
| men21_red564 | men21 | 52.93 | -51.96 |
| men21_red565 | men21 | 52.93 | -51.96 |
| men21_red566 | men21 | 52.93 | -51.96 |
| men21_red567 | men21 | 52.93 | -51.96 |
| men21_red568 | men21 | 52.93 | -51.96 |
| men21_red569 | men21 | 52.93 | -51.96 |
| men21_red570 | men21 | 52.93 | -51.96 |
| men21_red571 | men21 | 52.93 | -51.96 |
| men21_red572 | men21 | 52.93 | -51.96 |
| men21_red573 | men21 | 52.93 | -51.96 |
| men21_red574 | men21 | 52.93 | -51.96 |
| men21_red575 | men21 | 52.93 | -51.96 |
| men21_red576 | men21 | 52.93 | -51.96 |
| men21_red577 | men21 | 52.93 | -51.96 |
| men21_red578 | men21 | 52.93 | -51.96 |
| men21_red579 | men21 | 52.93 | -51.96 |
| men21_red580 | men21 | 53.36 | -52.23 |
| men21_red581 | men21 | 53.36 | -52.23 |
| men21_red582 | men21 | 53.36 | -52.23 |
| men21_red583 | men21 | 53.36 | -52.23 |
| men21_red584 | men21 | 53.36 | -52.23 |
| men21_red585 | men21 | 53.36 | -52.23 |
| men21_red586 | men21 | 53.36 | -52.23 |
| men21_red587 | men21 | 53.36 | -52.23 |
| men21_red588 | men21 | 53.36 | -52.23 |
| men21_red589 | men21 | 53.36 | -52.23 |
| men21_red590 | men21 | 53.36 | -52.23 |
| men21_red591 | men21 | 53.36 | -52.23 |
| men21_red592 | men21 | 53.36 | -52.23 |
| men21_red593 | men21 | 53.36 | -52.23 |
| men21_red594 | men21 | 53.36 | -52.23 |
| men21_red595 | men21 | 53.36 | -52.23 |
| men18_red743 | men18 | 64.56 | -58.52 |
| men18_red744 | men18 | 64.56 | -58.52 |
| men18_red745 | men18 | 64.56 | -58.52 |
| men19_red746 | men19 | 61.64 | -60.73 |
| men19_red747 | men19 | 61.64 | -60.73 |
| men19_red748 | men19 | 61.64 | -60.73 |
| men18_red749 | men18 | 62.69 | -61.28 |
| men18_red750 | men18 | 62.69 | -61.28 |
| men18_red751 | men18 | 62.69 | -61.28 |
| men18_red752 | men18 | 62.69 | -61.28 |
| men18_red753 | men18 | 63.95 | -59 |
| men18_red754 | men18 | 63.95 | -59 |
| men18_red755 | men18 | 63.95 | -59 |
| men20_red758 | men20 | 58.49 | -59.92 |
| men20_red760 | men20 | 58.49 | -59.92 |
| men20_red761 | men20 | 58.49 | -59.92 |
| men20_red762 | men20 | 59.62 | -60.63 |
| men20_red763 | men20 | 59.62 | -60.63 |
| men20_red767 | men20 | 61.46 | -60.34 |
| men20_red769 | men20 | 57.97 | -59.67 |
| men19_red771 | men19 | 60.48 | -63.84 |
| men20_red773 | men20 | 57.97 | -59.67 |
| men19_red774 | men19 | 60.48 | -63.84 |
| men19_red775 | men19 | 60.56 | -63.89 |
| men19_red776 | men19 | 60.56 | -63.89 |
| men19_red777 | men19 | 60.56 | -63.89 |
| men19_red778 | men19 | 60.56 | -63.89 |
| men19_red779 | men19 | 62.91 | -63.14 |
| men19_red780 | men19 | 62.91 | -63.14 |
| men19_red781 | men19 | 62.91 | -63.14 |
| men20_red782 | men20 | 57.97 | -59.67 |
| men18_red783 | men18 | 60.91 | -62.9 |
| men19_red784 | men19 | 62.91 | -62.9 |
| men18_red786 | men18 | 60.91 | -62.9 |
| men19_red787 | men19 | 62.71 | -62.03 |
| men18_red834 | men18 | 64.56 | -58.52 |
| men18_red835 | men18 | 64.56 | -58.52 |
| men19_red836 | men19 | 61.64 | -60.73 |
| men19_red837 | men19 | 61.64 | -60.73 |
| men18_red838 | men18 | 62.69 | -61.28 |
| men18_red839 | men18 | 62.69 | -61.28 |
| men18_red840 | men18 | 62.69 | -61.28 |
| men18_red841 | men18 | 62.69 | -61.28 |
| men18_red842 | men18 | 62.69 | -61.28 |
| men18_red843 | men18 | 62.69 | -61.28 |
| men18_red844 | men18 | 63.95 | -59 |
| men18_red846 | men18 | 63.95 | -59 |
| men18_red847 | men18 | 63.95 | -59 |
| men18_red848 | men18 | 63.95 | -59 |
| men18_red849 | men18 | 63.95 | -59 |
| men18_red850 | men18 | 63.95 | -59 |
| men18_red851 | men18 | 61.64 | -60.73 |
| men20_red852 | men20 | 57.97 | -59.67 |
| men20_red853 | men20 | 58.49 | -59.92 |
| men20_red854 | men20 | 58.49 | -59.92 |
| men20_red855 | men20 | 58.49 | -59.92 |
| men20_red856 | men20 | 59.62 | -60.63 |
| men20_red857 | men20 | 59.62 | -60.63 |
| men20_red858 | men20 | 59.62 | -60.63 |
| men20_red859 | men20 | 59.62 | -60.63 |
| men20_red860 | men20 | 59.62 | -60.63 |
| men20_red861 | men20 | 59.62 | -60.63 |
| men20_red862 | men20 | 59.62 | -60.63 |
| men20_red863 | men20 | 57.97 | -59.67 |
| men20_red864 | men20 | 59.62 | -60.63 |
| men20_red865 | men20 | 60.73 | -59.91 |
| men20_red868 | men20 | 61.46 | -60.34 |
| men19_red871 | men19 | 60.48 | -63.84 |
| men19_red872 | men19 | 60.56 | -63.89 |
| men20_red873 | men20 | 57.97 | -59.67 |
| men19_red874 | men19 | 62.91 | -63.14 |
| men19_red875 | men19 | 62.91 | -63.14 |
| men18_red876 | men18 | 60.91 | -62.9 |
| men19_red877 | men19 | 62.91 | -62.9 |
| men18_red878 | men18 | 60.91 | -62.9 |
| men19_red879 | men19 | 62.71 | -62.03 |
| men25_red880 | men25 | 45.11 | -57.56 |
| men25_red881 | men25 | 45.11 | -57.56 |
| men25_red882 | men25 | 45.11 | -57.56 |
| men25_red883 | men25 | 45.11 | -57.56 |
| men25_red884 | men25 | 45.11 | -57.56 |
| men25_red885 | men25 | 45.11 | -57.56 |
| men25_red886 | men25 | 45.11 | -57.56 |
| men25_red887 | men25 | 45.11 | -57.56 |
| men25_red888 | men25 | 45.11 | -57.56 |
| men25_red889 | men25 | 45.11 | -57.56 |
| men25_red890 | men25 | 45.11 | -57.56 |
| men25_red891 | men25 | 45.11 | -57.56 |
| men25_red892 | men25 | 45.11 | -57.56 |
| men25_red893 | men25 | 45.11 | -57.56 |
| men25_red894 | men25 | 45.11 | -57.56 |
| men25_red895 | men25 | 45.11 | -57.56 |
| men25_red896 | men25 | 45.11 | -57.56 |
| men25_red897 | men25 | 45.11 | -57.56 |
| men25_red898 | men25 | 45.11 | -57.56 |
| men25_red899 | men25 | 45.11 | -57.56 |
| men25_red900 | men25 | 45.11 | -57.56 |
| men25_red901 | men25 | 45.11 | -57.56 |
| men25_red902 | men25 | 45.11 | -57.56 |
| men25_red903 | men25 | 45.11 | -57.56 |
| men25_red904 | men25 | 45.11 | -57.56 |
| men25_red905 | men25 | 45.11 | -57.56 |
| men25_red906 | men25 | 45.11 | -57.56 |
| men25_red907 | men25 | 45.11 | -57.56 |
| men25_red908 | men25 | 45.11 | -57.56 |
| men25_red909 | men25 | 45.11 | -57.56 |
| men26_red910 | men26 | 47.2 | -57.3 |
| men26_red911 | men26 | 47.2 | -57.3 |
| men26_red912 | men26 | 47.2 | -57.3 |
| men26_red913 | men26 | 47.2 | -57.3 |
| men26_red914 | men26 | 47.2 | -57.3 |
| men26_red915 | men26 | 47.2 | -57.3 |
| men26_red916 | men26 | 47.2 | -57.3 |
| men26_red917 | men26 | 47.2 | -57.3 |
| men26_red918 | men26 | 47.2 | -57.3 |
| men26_red919 | men26 | 47.2 | -57.3 |
| men26_red920 | men26 | 47.2 | -57.3 |
| men26_red922 | men26 | 47.2 | -57.3 |
| men26_red923 | men26 | 47.2 | -57.3 |
| men26_red924 | men26 | 47.2 | -57.3 |
| men26_red925 | men26 | 47.2 | -57.3 |
| men26_red926 | men26 | 47.2 | -57.3 |
| men26_red927 | men26 | 47.2 | -57.3 |
| men26_red928 | men26 | 47.2 | -57.3 |
| men26_red929 | men26 | 47.2 | -57.3 |
| men26_red930 | men26 | 47.2 | -57.3 |
| men26_red931 | men26 | 47.2 | -57.3 |
| men26_red932 | men26 | 47.2 | -57.3 |
| men26_red933 | men26 | 47.2 | -57.3 |
| men26_red934 | men26 | 47.2 | -57.3 |
| men26_red935 | men26 | 47.2 | -57.3 |
| men26_red936 | men26 | 47.2 | -57.3 |
| men26_red937 | men26 | 47.2 | -57.3 |
| men26_red938 | men26 | 47.2 | -57.3 |
| men26_red939 | men26 | 47.2 | -57.3 |

**Table S2.** Filtering steps summary (Note: the number of the SNPs removed indicated is not exclusive of one filtering step).

|  | SNPs genotyped |
| --- | --- |
| STACKS CATALOGS | 72,933 |
| FILTERING STEPS | **SNPs removed** |
| Genotyped in more than 60% of the samples | 24,597 |
| MAF FILTERS |  |
| Global MAF > 0.01 | 12,869 |
| Local MAF > 0.05 | 6,532 |
| HWE FILTER |  |
| H_OBS_ < 0.6 | 13,411 |
| One SNP per locus  TOTAL | 10,639  **24,603** |

**Table S3.** List of selected explanatory variables contributing to the genomic variation present among 416 individuals genotyped as *S. mentella*. Variable name, cumulative adjusted *R^2^,* Akaike information criterion (AIC), F statistic, and probabilities (P-value) are indicated.

| **Variable** | **Cum Adjusted *R^2^*** | **AIC** | **F** | **P-value** |
| --- | --- | --- | --- | --- |
| Depth | 0.059220 | 3591.0 | 27.1235 | 0.002 |
| MEM2 | 0.068873 | 3587.7 | 5.2917 | 0.002 |
| MEM1 | 0.075884 | 3585.6 | 4.1335 | 0.002 |
| Y2013 | 0.078767 | 3585.3 | 2.2892 | 0.002 |
| MEM6 | 0.080758 | 3585.4 | 1.8902 | 0.002 |
| Y2008 | 0.082791 | 3585.4 | 1.9088 | 0.002 |
| MEM5 | 0.083509 | 3586.1 | 1.3206 | 0.002 |
| MEM7 | 0.084030 | 3586.8 | 1.2319 | 0.006 |
| MEM10 | 0.084542 | 3587.6 | 1.2276 | 0.008 |
| MEM8 | 0.084940 | 3588.4 | 1.1767 | 0.002 |

**Table S4.** List of selected explanatory variables contributing to the genomic variation present among 444 individuals genotyped as *S. fasciatus*. Variable name, cumulative adjusted *R^2^*, Akaike information criterion (AIC), F statistic, and probabilities (P-value) are indicated.

| **Variable** | **Cum Adjusted *R^2^*** | | **AIC** | **F** | **P-value** |
| --- | --- | --- | --- | --- | --- |
| MEM1 | 0.010295 | 3766.8 | | 5.6079 | 0.002 |
| Y2002 | 0.018989 | 3763.9 | | 4.9174 | 0.002 |
| Y2014 | 0.024662 | 3762.3 | | 3.5649 | 0.002 |
| MEM4 | 0.027317 | 3762.1 | | 2.2009 | 0.002 |
| MEM3 | 0.029487 | 3762.1 | | 1.9818 | 0.002 |
| MEM2 | 0.031331 | 3762.2 | | 1.8338 | 0.002 |
| Depth | 0.033071 | 3762.4 | | 1.7865 | 0.002 |
| Y2001 | 0.034464 | 3762.7 | | 1.6288 | 0.002 |
| MEM6 | 0.035740 | 3763.1 | | 1.5757 | 0.002 |
| Y2013 | 0.036695 | 3763.6 | | 1.4301 | 0.002 |
| MEM7 | 0.037643 | 3764.2 | | 1.4268 | 0.002 |
| MEM5 | 0.038476 | 3764.8 | | 1.3741 | 0.002 |
| MEM9 | 0.039158 | 3765.5 | | 1.2737 | 0.002 |

**Table S5.** Summary of the demographic models (model) tested between species and ecotypes (pop12): Akaike information criterion (AIC), deltaAIC and AICWeights. The best models are in bold.

| **pop12** | **Model** | **AIC** | **deltaAIC** | **AICWeights** |
| --- | --- | --- | --- | --- |
| **S. fasciatus vs S. mentella "deep"** | **SC2N** | **9587,95** | **0,0** | **1** |
| S. fasciatus vs S. mentella "deep" | IM2N2m | 9823,27 | 235,3 | 0 |
| S. fasciatus vs S. mentella "deep" | SC2N2m | 9863,53 | 275,6 | 0 |
| S. fasciatus vs S. mentella "deep" | SC2m | 10265,92 | 678,0 | 0 |
| S. fasciatus vs S. mentella "deep" | IM2N | 10462,94 | 875,0 | 0 |
| S. fasciatus vs S. mentella "deep" | AM2N | 10479,48 | 891,5 | 0 |
| S. fasciatus vs S. mentella "deep" | AM2m | 11186,09 | 1598,1 | 0 |
| S. fasciatus vs S. mentella "deep" | IM2m | 11193,63 | 1605,7 | 0 |
| S. fasciatus vs S. mentella "deep" | IM | 11456,19 | 1868,2 | 0 |
| S. fasciatus vs S. mentella "deep" | AM2N2m | 11748,46 | 2160,5 | 0 |
| S. fasciatus vs S. mentella "deep" | SC | 12390,04 | 2802,1 | 0 |
| S. fasciatus vs S. mentella "deep" | AM | 12394,58 | 2806,6 | 0 |
| S. fasciatus vs S. mentella "deep" | SI2N | 16455,88 | 6867,9 | 0 |
| S. fasciatus vs S. mentella "deep" | SI | 21831,66 | 12243,7 | 0 |
| **S. fasciatus vs S. mentalla "shallow"** | **SC2N** | **9651,98** | **0,0** | **1** |
| S. fasciatus vs S. mentalla "shallow" | SC2N2m | 9985,36 | 333,4 | 0 |
| S. fasciatus vs S. mentalla "shallow" | SC2m | 10373,31 | 721,3 | 0 |
| S. fasciatus vs S. mentalla "shallow" | IM2N2m | 10403,10 | 751,1 | 0 |
| S. fasciatus vs S. mentalla "shallow" | IM2N | 10923,31 | 1271,3 | 0 |
| S. fasciatus vs S. mentalla "shallow" | AM2N | 10929,67 | 1277,7 | 0 |
| S. fasciatus vs S. mentalla "shallow" | SC | 11576,79 | 1924,8 | 0 |
| S. fasciatus vs S. mentalla "shallow" | AM2m | 11664,23 | 2012,2 | 0 |
| S. fasciatus vs S. mentalla "shallow" | IM2m | 11670,55 | 2018,6 | 0 |
| S. fasciatus vs S. mentalla "shallow" | AM2N2m | 12546,30 | 2894,3 | 0 |
| S. fasciatus vs S. mentalla "shallow" | IM | 12548,57 | 2896,6 | 0 |
| S. fasciatus vs S. mentalla "shallow" | AM | 15419,40 | 5767,4 | 0 |
| S. fasciatus vs S. mentalla "shallow" | SI2N | 18808,35 | 9156,4 | 0 |
| S. fasciatus vs S. mentalla "shallow" | SI | 23410,89 | 13758,9 | 0 |
| **S. mentella "deep" vs "shallow"** | **SC2m** | **7426,08** | **0,0** | **1** |
| S. mentella "deep" vs "shallow" | SC2N2m | 7562,05 | 136,0 | 0 |
| S. mentella "deep" vs "shallow" | SC2N | 7596,19 | 170,1 | 0 |
| S. mentella "deep" vs "shallow" | IM2N2m | 7855,89 | 429,8 | 0 |
| S. mentella "deep" vs "shallow" | IM2N | 7897,32 | 471,2 | 0 |
| S. mentella "deep" vs "shallow" | AM2N | 7900,39 | 474,3 | 0 |
| S. mentella "deep" vs "shallow" | SC | 8202,37 | 776,3 | 0 |
| S. mentella "deep" vs "shallow" | IM2m | 8204,38 | 778,3 | 0 |
| S. mentella "deep" vs "shallow" | AM2m | 8310,51 | 884,4 | 0 |
| S. mentella "deep" vs "shallow" | AM2N2m | 8589,62 | 1163,5 | 0 |
| S. mentella "deep" vs "shallow" | IM | 8896,11 | 1470,0 | 0 |
| S. mentella "deep" vs "shallow" | AM | 8898,12 | 1472,0 | 0 |
| S. mentella "deep" vs "shallow" | SI2N | 10576,89 | 3150,8 | 0 |
